# Deuterosomal cells are the responsible lineage for multiciliogenesis in human airway differentiation

**DOI:** 10.1016/j.stemcr.2026.102860

**Published:** 2026-03-19

**Authors:** Haruka Yamaki, Satoshi Konishi, Koji Tamai, Naoyuki Sone, Senye Takahashi, Yifei Xu, Takahiro Tsuji, Hiroaki Ozasa, Takuya Yamamoto, Toyohiro Hirai, Kazuhiko Takeuchi, Shimpei Gotoh

**Affiliations:** 1 Center for iPS Cell Research and Application (CiRA), Kyoto University, Kyoto 606-8507, Japan; 2 Department of Respiratory Medicine, Graduate School of Medicine, Kyoto University, Kyoto 606-8507, Japan; 3 Department of Otorhinolaryngology, Head and Neck Surgery, Mie University Graduate School of Medicine, Tsu 514-8507, Japan; 4 Institute for the Advanced Study of Human Biology (WPI-ASHBi), Kyoto University, Kyoto 606-8501, Japan; 5 Medical-risk Avoidance Based on iPS Cells Team, RIKEN Center for Advanced Intelligence Project (AIP), Kyoto 606-8507, Japan; 6 Department of Otorhinolaryngology, Matsusaka Central General Hospital, Matsusaka 515-8566, Japan

**Keywords:** multiciliated cells, primary ciliary dyskinesia, deuterosomal cells, human airway epithelial differentiation, Cyclin O, CCNO, reduced generation of multiple motile cilia, RGMC, multiciliogenesis

## Abstract

Multiciliated cells (MCCs) are pivotal in airway defense via their motile cilia to eliminate inhaled pathogens and particles. Genetic variants in primary ciliary dyskinesia (PCD) disrupt ciliary function, resulting in chronic respiratory infections. The formation of MCCs requires centriole amplification mediated by non-membranous organelles called deuterosomes, whose regulatory mechanisms remain poorly characterized in humans. Single-cell transcriptomic analyses have identified “deuterosomal cells” (DCs), a transient cell population that emerges during multiciliogenesis. DCs are challenging to investigate owing to their scarcity. To elucidate the role of DCs, iPSC-derived airway epithelial cells were used to identify CD36 as a specific surface marker. Furthermore, iPSCs were established from a patient with PCD harboring *Cyclin O* (*CCNO*) variants, along with gene-corrected controls. Patient-derived iPSCs demonstrated defective MCC differentiation and aberrant DCs attributed to CCNO deficiency. This study provides a human iPSC-based platform for investigating the mechanisms underlying airway multiciliogenesis and PCD modeling.

## Highlights

• CD36 serves as a surface marker for isolating iPSC-derived deuterosomal cells (DCs)
• Altered DC identity in *CCNO* variants impairs multiciliated cells (MCCs) maturation
• *CCNO* variants disrupt DC-MCC pathway and promote expansion of aberrant precursors
• Human iPSC-derived DCs enable study of multiciliogenesis mechanisms

## Graphical abstract

**Figure:**
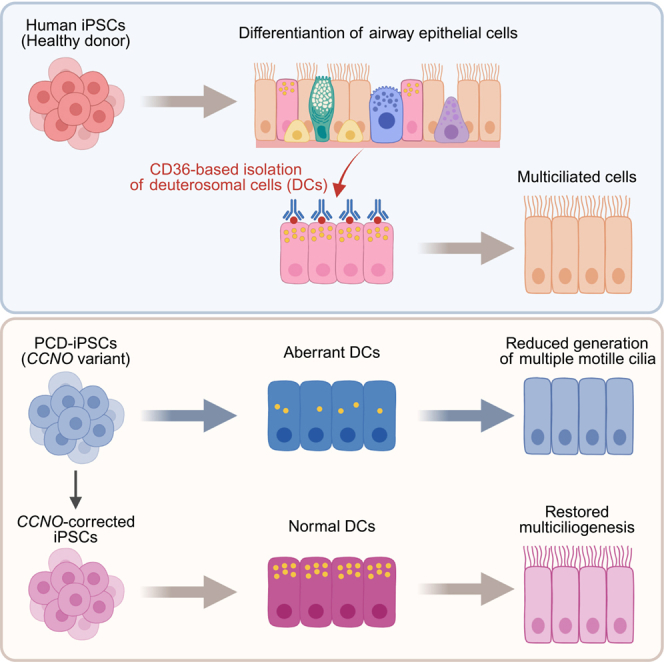

## Teaser

Gotoh, Konishi, and colleagues identify CD36 as a marker for isolating iPSC-derived deuterosomal cells (DCs). scRNA-seq demonstrates that *CCNO* variants associated with primary ciliary dyskinesia alter deuterosomal gene expression and disrupt DC-multiciliated cells (MCC) differentiation pathway, leading to aberrant MCC precursor accumulation. This study highlights the utility of iPSC-derived DCs for dissecting human multiciliogenesis dynamics and disease-associated differentiation defects.

## Introduction

Multiciliated cells (MCCs), bearing hundreds of apical motile cilia, clear inhaled pathogens, and particles via mucus transport. Primary ciliary dyskinesia (PCD) is a genetic disorder caused by defects in the structure, function, and biogenesis of motile cilia with pathogenic variants of numerous causative genes (Hannah et al., 2022). While most cases involve axonemal abnormalities, some patients exhibit reduced generation of multiple motile cilia (RGMC) due to variants in multiciliogenesis genes, such as *Cyclin O* (*CCNO*) and *MCIDAS* (Wallmeier et al., 2014; Boon et al., 2014). To generate basal bodies required for ciliogenesis, MCCs amplify centrioles via two distinct pathways: centriole duplication and *de novo* biogenesis mediated by deuterosomes (DS), specialized non-membranous organelles that serve as scaffolds. In vertebrates, most centrioles in the MCCs arise via a DS-dependent pathway (Zhao et al., 2013; Al Jord et al., 2014). CCNO is a critical regulator of DS-mediated centriole amplification, and pathogenic *CCNO* variants cause PCD with severe loss of motile cilia and mucociliary dysfunction (Wallmeier et al., 2014), consistent with *Ccno*-deficient mice (Funk et al., 2015). CCNO regulates the entry of MCC precursors into a specific cell cycle required for multiciliogenesis (Khoury Damaa et al., 2025). However, the regulatory mechanisms of DS pathway and the precise role of CCNO in MCC differentiation, particularly in human cells, are unclear. Single-cell RNA sequencing (scRNA-seq) of the human airway epithelium (AE) has identified deuterosomal cells (DCs), transient MCC precursors with high expression of DS-associated genes, as a transcriptionally distinct state during MCC differentiation (Revinski et al., 2018; Ruiz García et al., 2019). However, functional investigation of DCs remains challenging since they transiently emerge during development (He et al., 2022) or regeneration. Therefore, we used human-induced pluripotent stem cells (iPSCs), a renewable, genetically defined platform. Since human iPSC-derived AE cells (iAECs) have been used to model PCD (Sone et al., 2021; Hawkins et al., 2021; Brody et al., 2025), we hypothesized that an iPSC-based system could enable the investigation of early MCC differentiation. Herein, we used iAECs to investigate the molecular and functional properties of DCs during human MCC differentiation and determined how CCNO regulates this process and contributes to PCD pathogenesis.

## Results

### Identification of DCs in iAECs

**Figure 1:**
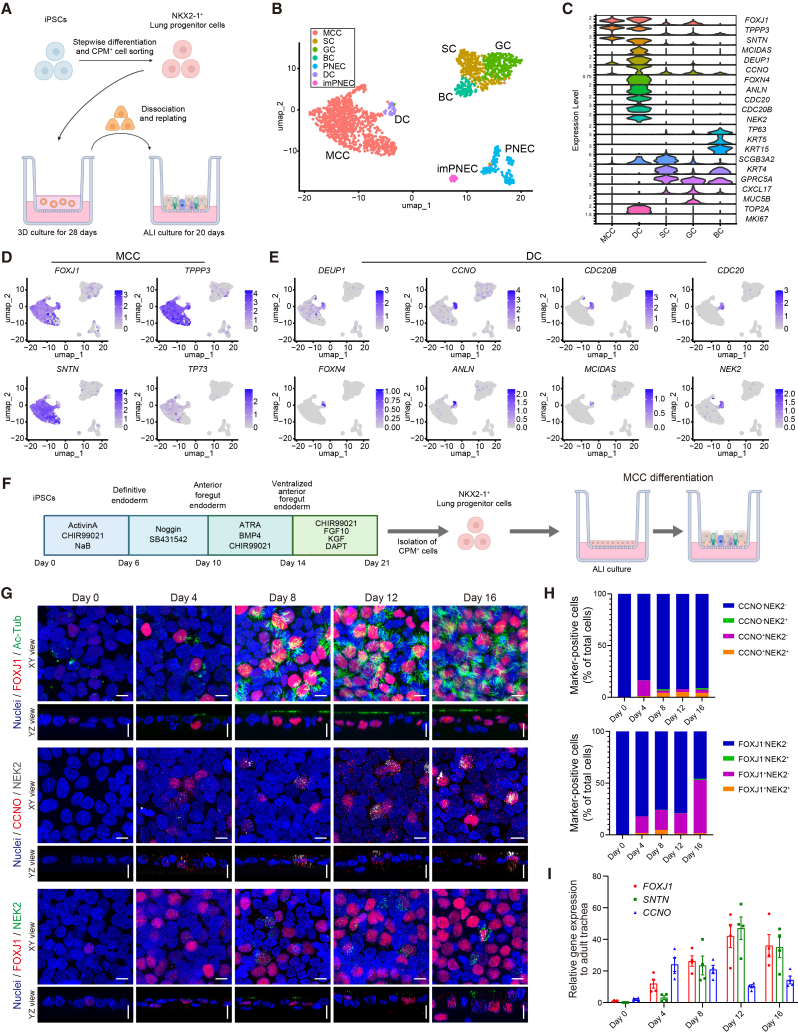
Identification and characterization of DCs in human iAECs (A) Schematic of iAEC generation via NKX2-1^+^ hLP isolation, followed by 3D and subsequent ALI culture. (B) UMAP of scRNA-seq of iAECs (201B7 iPSC line) on ALI day 20 following 3D culture. (C) Violin plots of major AE lineage markers of MCC, SC, GC, BC, and DC. (D and E) Feature plots of MCC (D) and DC (E) markers. (F) Schematic of iAEC generation via direct seeding of CPM^+^ hLPs onto ALI culture. (G) Time-course IFA of iAECs (B2-3 iPSC line) by direct ALI differentiation on ALI days 0, 4, 8, 12, and 16 (after DAPT addition). FOXJ1 (red) and Ac-Tub (green) in row 1; CCNO (red) and NEK2 (white) in row 2; and FOXJ1 (red) and NEK2 (green) in row 3. Maximum intensity z-projections and orthogonal views are shown for each time point and marker combination. Nuclei, Hoechst 33342 (blue). Scale bars, 10 μm. (H) Quantification of CCNO/NEK2- (top) and FOXJ1/NEK2-defined (bottom) cell populations during ALI differentiation (B2-3 iAECs) based on maximum intensity z-projections. Five randomly selected fields were quantified and averaged per differentiation. Stacked bar plots represent the mean percentage of each indicated cell population relative to the total number of nuclei from independent differentiations (*n* = 3). Numerical values (mean ± SEM) are provided in Table S1. (I) Time-course RT-qPCR of *FOXJ1*, *SNTN*, and *CCNO* expression in iAECs (B2-3) on ALI days 0, 4, 8, 12, and 16. Data are presented as mean ± SEM (*n* = 4, independent experiments).

We previously established a method for generating iAECs via iPSC-derived CPM^+^ lung progenitor cells (hLPs) (Gotoh et al., 2014) in three-dimensional (3D) culture (Figure 1A) (Konishi et al., 2016). This model recapitulates MCCs and pulmonary neuroendocrine cells (PNECs), while reproducing other major AE lineages, including basal cells (BCs), secretory cells (SCs), and goblet cells (GCs). Clustering of iAECs based on canonical marker gene expression revealed MCCs, BCs, SCs, GCs, and PNECs (Figures 1B, 1C, and S1A–S1E). We identified transcriptionally distinct DCs expressing DS-associated markers (*DEUP1*, *CCNO*, *CDC20B*, and *FOXN4*) and MCC markers with trajectories indicating MCCs derived from DCs (Figures 1C–1E, S1B, and S1F), as previously described in human AE (Revinski et al., 2018; Ruiz García et al., 2019). To analyze transient DCs, we replated hLPs onto an air-liquid interface (ALI) culture for multiciliogenesis, without 3D culture (Figure 1F), using a raised anti-CPM antibody (43A1) (Figure S1G). To capture the dynamics of MCC differentiation, we analyzed the cells on days 0, 4, 8, 12, and 16. Immunofluorescence analysis (IFA) revealed a gradual increase in the proportion of FOXJ1^+^ cells, reflecting MCC maturation. In contrast, the proportion of CCNO^+^ DCs peaked on day 4 and declined thereafter (Figures 1G, 1H, and S1H). At intermediate stages, NEK2^+^ cells were detected among the FOXJ1^+^ cells and a subset of CCNO^+^ cells (Figure 1G). Co-expression of DC markers, such as CCNO and SAS6, CCNO and FOXN4, and CDC20B and NEK2 was observed (Figure S1I), suggesting heterogeneity within the DC population. Consistently, RT-qPCR analysis showed that *CCNO* and MCC markers, such as *FOXJ1* and *SNTN*, exhibited distinct temporal expression patterns during differentiation, with *CCNO* expression peaking early, whereas *FOXJ1* expression increased progressively toward later stages (Figure 1I). These findings support DCs as transitional precursors preceding MCC maturation.

### CD36 enables isolation of iPSC-derived DCs

**Figure 2:**
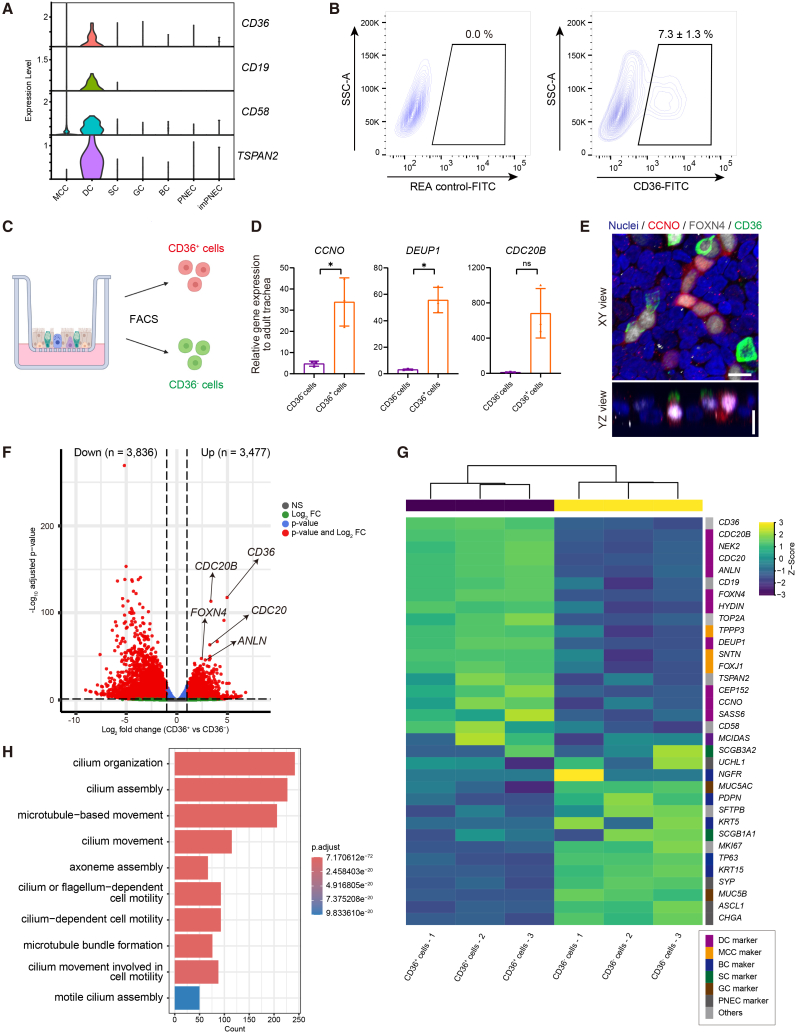
CD36 enables the isolation of human iDCs (A) Violin plots of candidate cell surface markers across clusters, with specific enrichment in the DC cluster, in iAECs (201B7) on ALI day 20 following 3D culture. (B) Flow cytometry of CD36 in iAECs (B2-3) generated by direct ALI differentiation on ALI day 12. Data are presented as mean ± SEM (*n* = 3, independent experiments). (C) Schematic of the flow cytometry-based strategy for sorting CD36^+^ and CD36^−^ cells from iAECs. (D) RT-qPCR of DS-associated genes in CD36^−^ and CD36^+^ cells sorted from iAECs (B2-3) on ALI day 12. Data are presented as mean ± SEM (*n* = 3, independent experiments). ^∗^*p* < 0.05; ns, not significant (paired *t* test). (E) IFA of CD36 in iAECs (B2-3) on ALI day 8, with co-staining for CCNO and FOXN4. CD36 (green), CCNO (red), and FOXN4 (white) are shown. Maximum intensity z-projection (top) and orthogonal view (bottom). Hoechst (blue). Scale bars, 10 μm. (F–H) Bulk RNA-seq analyses of CD36^+^ and CD36^−^ cells isolated from iAECs (B2-3) on ALI day 12 (*n* = 3, independent experiments). (F) Volcano plot for genes differentially expressed between CD36^+^ and CD36^−^ cells. Genes with |log_2_ fold change| > 1 and adjusted *p* value < 0.05 are considered significant (red). Selected DC marker genes are highlighted. (G) Heatmap of representative markers for major AE lineages in CD36^+^ and CD36^−^ cells. Gene expression values were scaled per row. Each column represents an individual sample replicate from the sorted populations. (H) GO analysis of biological process terms among differentially expressed genes between CD36^+^ and CD36^−^ cells. GO terms are ranked by adjusted *p* value, as indicated by the color. Bar length indicates the number of associated genes.

To characterize DCs in iAECs (iDCs), we analyzed scRNA-seq data to identify iDC-specific surface markers. Among four candidate surface markers for fluorescence-activated cell sorting (FACS) (Figure 2A), only CD36 exhibited reproducible surface expression in a subset of iAECs (Figures 2B and S2A–S2C). We therefore focused on subsequent analyses of the CD36^+^ population, which comprised a small fraction of ALI-cultured iAECs, and isolated CD36^+^ and CD36^−^ cell populations (Figure 2C). RT-qPCR revealed enrichment of DC markers (*CCNO*, *DEUP1*, and *CDC20B*) in CD36^+^ cells compared with CD36^−^ cells derived from three donor-derived iPSCs (Figures 2D, S2D, and S3E), and CD36 was localized to the apical side of cells expressing the DC markers CCNO and FOXN4 (Figure 2E). Bulk RNA-seq analyses revealed a clear separation between CD36^+^ and CD36^−^ cells in principle component analysis (PCA) (Figure S2E). Differential gene expression analysis revealed widespread transcriptional differences between CD36^+^ and CD36^−^ cells, with enrichment of DC markers in CD36^+^ cells (Figure 2F). Heatmap analysis also revealed high expression of DC markers in CD36^+^ cells, accompanied by enriched MCC markers and low expression of other AE lineage markers (Figure 2G). Gene ontology (GO) analysis of the upregulated genes in CD36^+^ cells showed enrichment in multiciliogenesis-related process (Figure 2H). These findings suggest that CD36^+^ cells exhibit DC identity and are committed to the MCC lineage, with minimal contributions from other epithelial lineages. Functionally, CD36-based cell sorting followed by re-plating under ALI conditions showed that CD36^+^ cells gave rise to MCC marker-positive cells, whereas CD36^−^ cells exhibited limited MCC differentiation (Figures S2F and S2G). To assess the relevance of CD36 expression in native human AE, we reanalyzed public human lung scRNA-seq datasets, which showed little to no CD36 expression in DCs and MCCs of adult lungs and fetal lungs (Figure S2H). Consistently, IFA of adult and fetal lung tissues identified DC marker-positive cells without co-expression of CD36 (Figure S2I). These results indicate that CD36 expression is not a prominent feature of DCs in native human AE, but rather emerges in iAECs, where it enables their isolation and functional analysis *in vitro*.

### Generation and comparison between *CCNO*-variant and gene-corrected iPSCs

**Figure 3:**
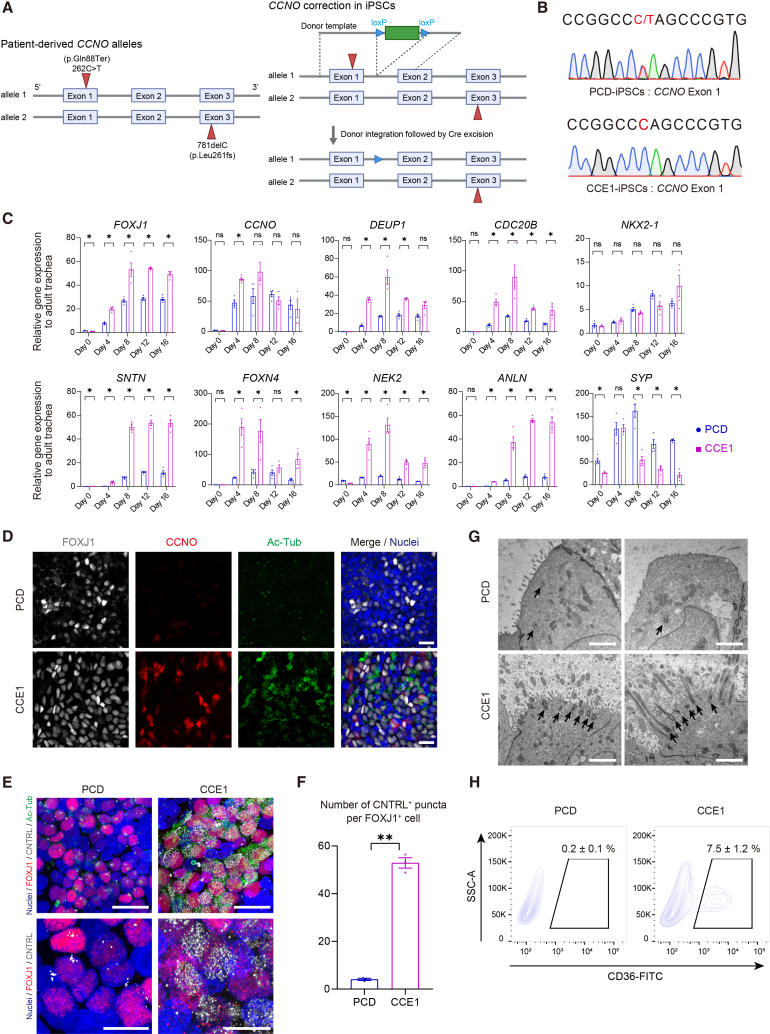
Generation and analysis of iPSCs from a patient with PCD harboring *CCNO* variants and their gene-corrected iPSCs (A) Schematic of compound heterozygous *CCNO* variants identified in the patient with PCD (left), and the gene editing strategy used to generate isogenic CCE1-iPSCs (right). (B) Sanger sequencing of the *CCNO* exon 1 region in PCD- and CCE1-iPSCs, showing successful correction of the nonsense variant. The heterozygous C>T variant in PCD-iPSCs and the corrected wild-type C in CCE1-iPSCs are indicated in red. (C) Time-course RT-qPCR of DS-associated and selected AE lineage markers (MCCs, hLPs, and PNECs) during iAEC differentiation in PCD- and CCE1-iAECs (*n* = 4 replicates at each time point). Data are presented as mean ± SEM. ^∗^*p* < 0.05 (Mann-Whitney *U* test). (D) Maximum intensity z-projections of IFA in PCD- and CCE1-iAECs on ALI day 8. FOXJ1 (white), CCNO (red), and Ac-Tub (green) are shown. Hoechst (blue). Scale bars, 20 μm. (E) IFA for CNTRL in PCD- and CCE1-iAECs on ALI day 16. Maximum intensity z-projections at low (top images) and high (bottom images) magnification. Top images show FOXJ1 (red), CNTRL (white), and Ac-Tub (green); bottom images show FOXJ1 (red) and CNTRL (white). Hoechst (blue). Scale bars, 20 μm (top images); 10 μm (bottom images). (F) Quantification of CNTRL^+^ puncta per FOXJ1^+^ cell based on maximum intensity z-projections. Five randomly selected fields were analyzed per differentiation. Data are presented as mean ± SEM (*n* = 3, independent differentiations). ^∗∗^*p* < 0.01 (paired *t* test). (G) TEM of PCD- and CCE1-iAECs on day 41 of 3D culture. Arrows indicate basal bodies. Scale bars, 2 μm. (H) Flow cytometry of CD36^+^ cell proportions in PCD- and CCE1-iAECs on ALI days 4–16 (*n* = 3 for PCD-iAECs; *n* = 6 for CCE1-iAECs). Data are presented as mean ± SEM.

Next, we generated iPSCs from a 5-year-old patient with PCD harboring compound heterozygous *CCNO* variants: a nonsense variant in exon 1 (allele 1: c.262C>T; p.Gln88Ter) and a frameshift deletion in exon 3 (allele 2: c.781delC; p.Leu261fs) (Xu et al., 2024), hereafter termed as PCD-iPSCs, and established their isogenic control iPSCs by CRISPR/Cas9-mediated correction of the exon 1 variant for comparative analysis (Figures 3A and 3B), referred to as CCE1-iPSCs (*CCNO*-corrected exon 1). Both iPSC lines showed normal karyotypes (Figure S3A) and matched short tandem repeat (STR) profiles with the patient’s peripheral blood mononuclear cells (PCD-PBMCs) (Figure S3B). The expression of pluripotency markers and trilineage differentiation were validated in PCD-iPSCs (Figure S3C). We differentiated both lines into CPM^+^ hLPs, with no apparent difference in the induction efficiency of NKX2-1^+^ cells (Figure S3D), and subsequently differentiated them into iAECs in ALI culture. *FOXJ1* and the motile cilia-specific marker *SNTN* were reduced in PCD-iAECs (Figure 3C). *CCNO* mRNA levels were not altered at most time points, except on day 4, possibly due to escape from nonsense-mediated decay by at least one of the variants. In contrast, the expression of several other DS-associated genes (Figure 3C) was decreased, suggesting impaired *CCNO* function. Additionally, no significant difference was observed in *NKX2-1*, a marker of hLPs, whereas the PNEC marker *SYP* was increased in PCD-iAECs. CCNO and acetylated tubulin (Ac-Tub) were decreased in PCD-iAECs, whereas FOXJ1 remained comparable between the two lines (Figure 3D). The number of CNTRL^+^ puncta was decreased in PCD-iAECs (Figures 3E and 3F). Transmission electron microscopy (TEM) analysis showed that PCD-iAECs exhibited apical surfaces covered with microvilli but lacked recognizable basal bodies and cilia, indicating a defect in multiciliogenesis. In contrast, CCE1-iAECs had well-organized motile cilia with identifiable basal bodies anchored to the apical membrane (Figure 3G). CD36^+^ cells were undetectable in PCD-iAECs, whereas CD36^+^ cells with enriched DS-associated gene expression were detected in CCE1-iAECs (Figures 3H and S3E). As an independent validation, correction of the exon 3 variant in an isogenic iPSC line (CCE3-iPSCs; *CCNO*-corrected exon 3) showed a normal karyotype (Figure S3A) and STR profiles (Figure S3B). Consistently, CCE3-iAECs restored CCNO expression, thereby regaining the DC/MCC dynamics and multiciliogenesis, as confirmed by RT-qPCR and IFA (Figures S3F and S3G).

### scRNA-seq reveals the role of CCNO in DC-mediated multiciliogenesis

**Figure 4:**
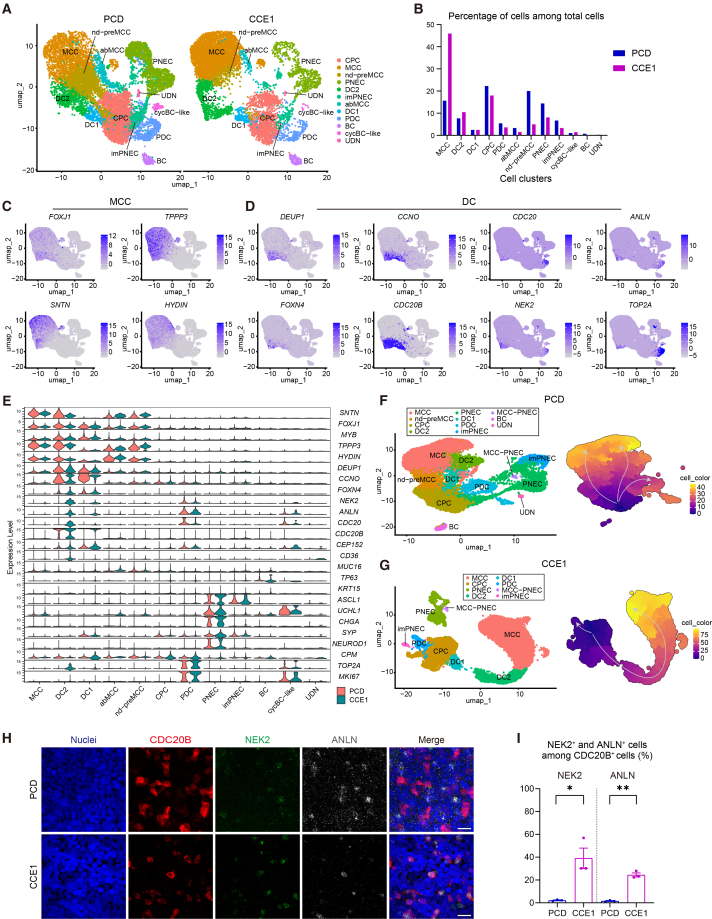
scRNA-seq comparative analysis between PCD- and CCE1-iAECs reveals CCNO-dependent impairment of DC-mediated multiciliogenesis (A) UMAP of the integrated scRNA-seq, shown separately for PCD-iAECs (left) and CCE1-iAECs (right), on ALI day 8. imPNEC, immature PNEC; UDN, undetermined. (B) Proportion of each cluster among total cells in PCD- and CCE1-iAECs in the scRNA-seq from ALI day 8 cultures. (C and D) Feature plots of representative MCC (C) and DC (D) markers. (E) Violin plots of major AE and DC markers in PCD- and CCE1-iAECs. (F and G) Left: UMAP of pre-integration scRNA-seq derived from PCD-iAECs (F) and CCE1-iAECs (G). Right: Visualization of MCC/DC-related clusters in each condition along the pseudotime trajectory based on pre-integration data, color-coded by pseudotime values. (H) IFA for CDC20B (red), NEK2 (green), and ANLN (white) in PCD- and CCE1-iAECs on ALI day 8. Maximum intensity z-projections are shown. Hoechst (blue). Scale bars, 20 μm. (I) Quantification of NEK2^+^ (left) and ANLN^+^ (right) cells among CDC20B^+^ cells in PCD- and CCE1-iAECs. Five randomly selected fields were analyzed per condition. Data are presented as mean ± SEM (*n* = 3, independent differentiations). ^∗^*p* < 0.05; ^∗∗^*p* < 0.01 (paired *t* test).

We performed scRNA-seq comparative analysis of PCD- and CCE1-iAECs on day 8 after starting MCC induction, when DCs were enriched according to the RT-qPCR and IFA results (Figures 1G and 3C). UMAP of the integrated scRNA-seq datasets identified 12 distinct clusters and showed an increased representation of cells in intermediate AE differentiation states relative to the 3D-ALI-derived dataset (Figures 1B and 4A, 4C–4E, S4A, and S4B). DCs constituted 15.6% and 16.5% of total cells in PCD- and in CCE1-iAECs, respectively (Figure 4B). We identified two populations representing undifferentiated states. *CPM*-expressing cells with low levels of DC, MCC, and PNEC markers were defined as common progenitor cells (CPCs), reflecting an early stage of AE differentiation from CPM^+^ hLPs. Cycling basal cells (cycBCs) were annotated based on the expression of BC- and proliferation-associated genes, as previously described (Plasschaert et al., 2018). DCs were further subdivided into DC1, DC2, and proliferative DCs (PDCs) subpopulations. DC1 was characterized by low *SNTN*, suggesting a less differentiated DC state. DC2 expressed *SNTN* and *TOP2A*, indicating a proliferating transitional stage between DC1 and MCCs. PDCs expressed proliferation- and DS-associated genes, suggesting a proliferative DC subset. In the DC2 cluster, *NEK2*, *ANLN*, and *CDC20* levels were lower in PCD-iAECs than in CCE1-iAECs, implying that these genes may act downstream of *CCNO* to regulate DC maturation. The two clusters expanded more in PCD-iAECs than in CCE1-iAECs. One cluster lacked the expression of DS-associated genes and *SNTN* and was defined as non-deuterosomal precursor MCCs (nd-preMCCs), while the other expressed *FOXJ1* but exhibited low *SNTN* with minimal expression of DS-associated genes and structural genes for motile cilia and was defined as aberrant MCCs (abMCCs). The proportion of MCCs in PCD-iAECs decreased (15.6%) compared with CCE1-iAECs (45.9%), indicating that the expansion of nd-preMCCs and abMCCs was associated with defective MCC differentiation in PCD-iAECs. In addition, PNEC populations modestly increased in PCD-iAECs (Figure 4B). To assess the developmental maturity of these AE lineages, we compared module scores of DCs, MCCs, and PNECs between iAECs and adult and fetal human lung scRNA-seq datasets (Figures S4C and S4D). DC2 cells derived from CCE1-iPSCs showed module scores comparable to adult lung DCs, whereas those from PCD-iPSCs were closer to fetal lung DCs. MCCs derived from CCE1-iPSCs exhibited module scores comparable to both fetal and adult MCCs, while those derived from PCD-iPSCs showed a slightly broader score distribution. The iPSC-derived PNECs were more closely aligned with fetal than adult PNECs. In CCE1-iAECs, pseudotime analysis revealed two differentiation trajectories: one proceeding from CPCs through DC1 and DC2 to MCCs and another branching from CPCs to PDCs (Figure 4G). Among these, the CPC-DC1-DC2-MCC pathway was predominant, consistent with progressive upregulation of MCC-associated genes (Figure S4E). In contrast, PCD-iAECs displayed an additional trajectory from CPCs to MCCs via nd-preMCCs, bypassing the DC states (Figures 4F and S4F). Integration of the two datasets supported this shift: the proportion of cells following the CPC-DC1-DC2-MCC trajectory was reduced in PCD-iAECs, whereas more cells followed the CPC-nd-preMCC-MCC trajectory. RNA velocity analysis independently confirmed these findings. CCE1-iAECs exhibited directional flow from DC1 to DC2 to MCCs, with bidirectional vectors between DC1 and DC2, consistent with a transient deuterosomal state. This dynamic was largely absent in PCD-iAECs, which showed reduced DC-associated flux and increased DC-bypassing trajectories with emergence of nd-preMCCs toward MCCs (Figure S4G). In IFA, the cells co-expressing CDC20B, NEK2, and ANLN in CCE1-iAECs were consistent with the transcriptional profile of DC2 (Figure 4H). In contrast, NEK2 and ANLN in PCD-iAECs were mainly expressed in CDC20B^−^ cells, suggesting a shift from a normal DC2 identity to an aberrant DC2-like phenotype, along with a relative increase in PDCs. The proportions of NEK2^+^ and ANLN^+^ cells among CDC20B^+^ cells were markedly reduced in PCD-iAECs compared with CCE1-iAECs (NEK2: 2.16 ± 0.39% vs. 39.1 ± 8.9%; ANLN: 1.60 ± 0.37% vs. 24.5 ± 1.8%) (Figure 4I). These results suggest that CCNO dysfunction disrupts DC-mediated multiciliogenesis and promotes an aberrant DC-bypassing differentiation.

## Discussion

This study identified DCs in human iAECs. To date, studies on the DS pathway and DCs have predominantly been conducted in Xenopus (Zhao et al., 2013; Wallmeier et al., 2014; Revinski et al., 2018) and mouse models (Mercey et al., 2019). Human studies on DC biology remain limited (Revinski et al., 2018; Ruiz García et al., 2019). Our human iPSC-based system provides stable access to DCs, enabling analysis of regulatory transitions during human multiciliogenesis. Integration of our scRNA-seq data with the public adult and fetal human lung atlases enabled a quantitative assessment of model fidelity and developmental maturity (Figure S4C). DC2 cells derived from CCE1-iPSCs closely resembled adult human lung DCs, whereas MCCs derived from CCE1-iPSCs exhibited almost equivalent module scores to fetal and adult MCCs. In contrast, MCCs derived from PCD-iPSCs showed a slightly broader score distribution, suggesting that multiciliogenesis is impaired due to CCNO deficiency. Together, these findings demonstrate that the iAECs capture lineage-specific transcriptional programs while revealing disease-associated delays in DC maturation and MCC differentiation. We isolated DCs from iAECs using CD36 as a surface antigen, which would facilitate downstream applications to study multiciliogenesis *in vitro*. While non-human models have linked CCNO, MCIDAS, and DEUP1 to DS regulation (Ma et al., 2014; Zhao et al., 2013; Funk et al., 2015), its regulation in humans remains unclear. We identified *ANLN*, *NEK2*, and *CDC20* as CCNO-downregulated genes in DC2 of PCD-iAECs, suggesting a transcriptional axis required for DS-mediated centriole amplification, consistent with recent findings implicating CCNO in a noncanonical cell-cycle program during MCC differentiation (Khoury Damaa et al., 2025). The difference between transcript- and protein-level measurements for DS-associated genes reflects cell-state-specific regulation during DS maturation. Reduced *NEK2* and *ANLN* expression was confined to the late DC2 state in PCD-iAECs, whereas IFA did not reveal a global reduction in protein abundance across the entire epithelial population. In the present study, PCD-iAECs with *CCNO* variants showed an increase in PDCs co-expressing proliferation- and DS-related genes, suggesting an impaired exit from the proliferative state and failure to complete differentiation. We speculate that this aberrant pathway reflects compensatory use of the mother centriole-dependent pathway, which may be insufficient for full MCC maturation. In addition to impaired DC-mediated multiciliogenesis, CCNO deficiency altered AE lineage balance within this *in vitro* system, including a modest increase in PNEC populations. Given that *CCNO* was not expressed in immature PNECs (imPNECs), this change likely reflects an indirect consequence of defective MCC differentiation. Together, these findings indicate that, within the context of CCNO-associated PCD, disruption of the DS-dependent DC-to-MCC differentiation pathway underlies the primary defect, whereas changes in other epithelial lineages arise as secondary effects of impaired multiciliogenesis.

## Methods

### Ethics

The generation and use of patient-derived iPSCs and the use of human lung tissue were approved by the Ethics Committee of the Graduate School and Faculty of Medicine and CiRA, Kyoto University (R91/G259 and R1009/G1074). Written informed consent was obtained from the patients’ legal guardians.

### Statistical analysis

Statistical analyses of the data were performed using GraphPad Prism version 8 (GraphPad Software) and R. Data are presented as mean ± standard error of the mean (SEM). Comparisons between the two groups were conducted using the Mann-Whitney *U* test or paired *t* test, as appropriate. Statistical significance was set at *p* < 0.05. The statistical tests used are described in the figure legends.

## Resource availability

### Lead contact

Requests for further information and resources should be directed to and will be fulfilled by the lead contact, Shimpei Gotoh (gotoh.shimpei.5m@kyoto-u.ac.jp).

### Materials availability

Materials used in this study are available upon request under a completed material transfer agreement.

### Data and code availability

Bulk and single-cell RNA-seq data generated in this study have been deposited in the Gene Expression Omnibus (GEO: GSE299246 and GSE299245), and in the DNA DataBank of Japan (DDBJ: J-DS000923). The data will be made publicly available upon publication.

## Acknowledgments

We thank S. Sakurai and K. Deguchi of the genome analysis group in the CiRA Common Equipment Management Office for RNA-seq library preparation and sequencing with analysis; S. Ikeo and Y. Yamamoto for supporting the generation of anti-CPM antibody in the past; and K. Okamoto-Furuta and T. Katsuno at the Division of Electron Microscopic Study, Center for Anatomical Studies, 10.13039/501100005683Kyoto University, for supporting electron microscopy. This study was funded by 10.13039/501100001691JSPS 10.13039/501100001691KAKENHI (JP22H03077, JP23K24338, JP25K12809, and JP25K02660); 10.13039/100009619AMED (JP19ek0109410, JP23bm1323001, and JP25bk0104190); the 10.13039/100019432Fujiwara Memorial Foundation; the 10.13039/100007428Naito Foundation; and the iPS Cell Research Fund for CiRA at Kyoto University. Schematic illustrations in this manuscript were created using BioRender.com.

## Author contributions

Conceptualization, H.Y., S.K., and S.G.; methodology, H.Y., S.K., Ko.T., N.S., S.T., and S.G.; validation, S.K. and N.S.; formal analysis, H.Y., S.K., T.Y., and S.G.; investigation, H.Y., S.K., Ko.T., S.T., and S.G.; resources, Y.X., T.T., H.O., Ka.T., and S.G.; writing, H.Y., S.K., and S.G.; supervision, T.H. and Ka.T.

## Declaration of interests

S.G. is the founder and shareholder of HiLung, Inc. S.K. and S.G. are the inventors of Kyoto University’s patents related to the method of generating AE: WO2016148307A1.

## Supplemental information

Document S1. Figures S1–S4, Tables S1 and S2, and supplemental experimental procedures

Table S3. Predicted off-target sites for the CRISPR/Cas9 sgRNA used for CCE1-iPSC generation

Table S4. Primers used for validation of predicted off-target sites

Table S5. Antibodies and immunostaining conditions

Table S6. Primers used for RT-qPCR

Document S2. Article plus supplemental information
