# Supplementary material for: Deuterosomal cells are the responsible lineage for multiciliogenesis in human airway differentiation
Source: Stem Cell Reports. 2026 Mar 19;21(4):102860. doi: 10.1016/j.stemcr.2026.102860 (PMC13083802; doi:10.1016/j.stemcr.2026.102860)
Supplement: Document S2. Article plus supplemental information [file mmc6.pdf]

STEM CELL REPORTS ISSCR

Report

OPEN ACCESS

Deuterosomal cells are the responsible lineage for multiciliogenesis in human airway differentiation

Haruka Yamaki,[1,2] Satoshi Konishi,[1,*] Koji Tamai,[1] Naoyuki Sone,[1] Senye Takahashi,[1] Yifei Xu,[1,3] Takahiro Tsuji,[2] Hiroaki Ozasa,[2] Takuya Yamamoto,[1,4,5] Toyohiro Hirai,[2] Kazuhiko Takeuchi,[3,6] and Shimpei Gotoh[1,7,*]

[1]Center for iPS Cell Research and Application (CiRA), Kyoto University, Kyoto 606-8507, Japan

[2]Department of Respiratory Medicine, Graduate School of Medicine, Kyoto University, Kyoto 606-8507, Japan

[3]Department of Otorhinolaryngology, Head and Neck Surgery, Mie University Graduate School of Medicine, Tsu 514-8507, Japan

[4]Institute for the Advanced Study of Human Biology (WPI-ASHBi), Kyoto University, Kyoto 606-8501, Japan

[5]Medical-risk Avoidance Based on iPS Cells Team, RIKEN Center for Advanced Intelligence Project (AIP), Kyoto 606-8507, Japan

[6]Department of Otorhinolaryngology, Matsusaka Central General Hospital, Matsusaka 515-8566, Japan

[7]Lead contact

*Correspondence: satoshi.konishi@cira.kyoto-u.ac.jp (S.K.), gotoh.shimpei.5m@kyoto-u.ac.jp (S.G.)

https://doi.org/10.1016/j.stemcr.2026.102860

SUMMARY

Multiciliated cells (MCCs) are pivotal in airway defense via their motile cilia to eliminate inhaled pathogens and particles. Genetic variants in primary ciliary dyskinesia (PCD) disrupt ciliary function, resulting in chronic respiratory infections. The formation of MCCs requires centriole amplification mediated by non-membranous organelles called deuterosomes, whose regulatory mechanisms remain poorly characterized in humans. Single-cell transcriptomic analyses have identified "deuterosomal cells" (DCs), a transient cell population that emerges during multiciliogenesis. DCs are challenging to investigate owing to their scarcity. To elucidate the role of DCs, iPSC-derived airway epithelial cells were used to identify CD36 as a specific surface marker. Furthermore, iPSCs were established from a patient with PCD harboring *Cyclin O* (*CCNO*) variants, along with gene-corrected controls. Patient-derived iPSCs demonstrated defective MCC differentiation and aberrant DCs attributed to CCNO deficiency. This study provides a human iPSC-based platform for investigating the mechanisms underlying airway multiciliogenesis and PCD modeling.

INTRODUCTION

Multiciliated cells (MCCs), bearing hundreds of apical motile cilia, clear inhaled pathogens, and particles via mucus transport. Primary ciliary dyskinesia (PCD) is a genetic disorder caused by defects in the structure, function, and biogenesis of motile cilia with pathogenic variants of numerous causative genes (Hannah et al., 2022). While most cases involve axonemal abnormalities, some patients exhibit reduced generation of multiple motile cilia (RGMC) due to variants in multiciliogenesis genes, such as *Cyclin O* (*CCNO*) and *MCIDAS* (Wallmeier et al., 2014; Boon et al., 2014). To generate basal bodies required for ciliogenesis, MCCs amplify centrioles via two distinct pathways: centriole duplication and *de novo* biogenesis mediated by deuterosomes (DS), specialized non-membranous organelles that serve as scaffolds. In vertebrates, most centrioles in the MCCs arise via a DS-dependent pathway (Zhao et al., 2013; Al Jord et al., 2014). CCNO is a critical regulator of DS-mediated centriole amplification, and pathogenic *CCNO* variants cause PCD with severe loss of motile cilia and mucociliary dysfunction (Wallmeier et al., 2014), consistent with *Ccno*-deficient mice (Funk et al., 2015). CCNO regulates the entry of MCC precursors into a specific cell cycle required for multiciliogenesis (Khoury Damaa et al., 2025). However, the regulatory mechanisms of DS pathway and the precise role of CCNO in MCC differentiation, particularly in human cells, are unclear. Single-cell RNA sequencing (scRNA-seq) of the human airway epithelium (AE) has identified deuterosomal cells (DCs), transient MCC precursors with high expression of DS-associated genes, as a transcriptionally distinct state during MCC differentiation (Revinski et al., 2018; Ruiz García et al., 2019). However, functional investigation of DCs remains challenging since they transiently emerge during development (He et al., 2022) or regeneration. Therefore, we used human-induced pluripotent stem cells (iPSCs), a renewable, genetically defined platform. Since human iPSC-derived AE cells (iAECs) have been used to model PCD (Sone et al., 2021; Hawkins et al., 2021; Brody et al., 2025), we hypothesized that an iPSC-based system could enable the investigation of early MCC differentiation. Herein, we used iAECs to investigate the molecular and functional properties of DCs during human MCC differentiation and determined how CCNO regulates this process and contributes to PCD pathogenesis.

RESULTS

Identification of DCs in iAECs

We previously established a method for generating iAECs via iPSC-derived CPM$^+$ lung progenitor cells (hLPs) (Gotoh et al., 2014) in three-dimensional (3D) culture

© 2026 The Author(s).
Published by Elsevier Inc. on behalf of International Society for Stem Cell Research.
This is an open access article under the CC BY license (http://creativecommons.org/licenses/by/4.0/).

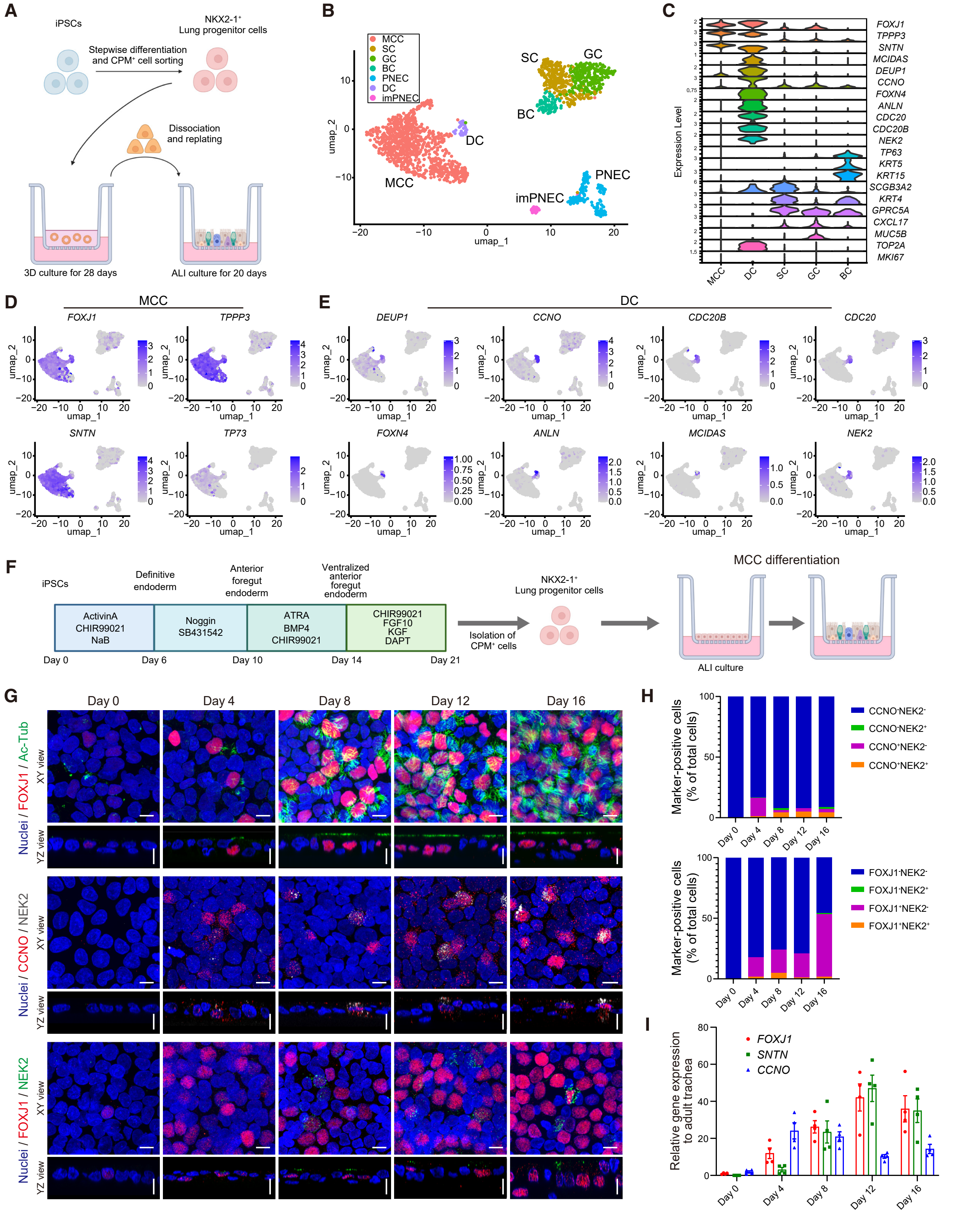

(legend on next page)

(Figure 1A) (Konishi et al., 2016). This model recapitulates MCCs and pulmonary neuroendocrine cells (PNECs), while reproducing other major AE lineages, including basal cells (BCs), secretory cells (SCs), and goblet cells (GCs). Clustering of iAECs based on canonical marker gene expression revealed MCCs, BCs, SCs, GCs, and PNECs (Figures 1B, 1C, and S1A–S1E). We identified transcriptionally distinct DCs expressing DS-associated markers (*DEUP1*, *CCNO*, *CDC20B*, and *FOXN4*) and MCC markers with trajectories indicating MCCs derived from DCs (Figures 1C–1E, S1B, and S1F), as previously described in human AE (Revinski et al., 2018; Ruiz García et al., 2019). To analyze transient DCs, we replated hLPs onto an air-liquid interface (ALI) culture for multiciliogenesis, without 3D culture (Figure 1F), using a raised anti-CPM antibody (43A1) (Figure S1G). To capture the dynamics of MCC differentiation, we analyzed the cells on days 0, 4, 8, 12, and 16. Immunofluorescence analysis (IFA) revealed a gradual increase in the proportion of FOXJ1$^+$ cells, reflecting MCC maturation. In contrast, the proportion of CCNO$^+$ DCs peaked on day 4 and declined thereafter (Figures 1G, 1H, and S1H). At intermediate stages, NEK2$^+$ cells were detected among the FOXJ1$^+$ cells and a subset of CCNO$^+$ cells (Figure 1G). Co-expression of DC markers, such as CCNO and SAS6, CCNO and FOXN4, and CDC20B and NEK2 was observed (Figure S1I), suggesting heterogeneity within the DC population. Consistently, RT-qPCR analysis showed that *CCNO* and MCC markers, such as *FOXJ1* and *SNTN*, exhibited distinct temporal expression patterns during differentiation, with *CCNO* expression peaking early, whereas *FOXJ1* expression increased progressively toward later stages (Figure 1I). These findings support DCs as transitional precursors preceding MCC maturation.

CD36 enables isolation of iPSC-derived DCs

To characterize DCs in iAECs (iDCs), we analyzed scRNA-seq data to identify iDC-specific surface markers. Among four candidate surface markers for fluorescence-activated cell sorting (FACS) (Figure 2A), only CD36 exhibited reproducible surface expression in a subset of iAECs (Figures 2B and S2A–S2C). We therefore focused on subsequent analyses of the CD36$^+$ population, which comprised a small fraction of ALI-cultured iAECs, and isolated CD36$^+$ and CD36$^-$ cell populations (Figure 2C). RT-qPCR revealed enrichment of DC markers (*CCNO*, *DEUP1*, and *CDC20B*) in CD36$^+$ cells compared with CD36$^-$ cells derived from three donor-derived iPSCs (Figures 2D, S2D, and S3E), and CD36 was localized to the apical side of cells expressing the DC markers CCNO and FOXN4 (Figure 2E). Bulk RNA-seq analyses revealed a clear separation between CD36$^+$ and CD36$^-$ cells in principle component analysis (PCA) (Figure S2E). Differential gene expression analysis revealed widespread transcriptional differences between CD36$^+$ and CD36$^-$ cells, with enrichment of DC markers in CD36$^+$ cells (Figure 2F). Heatmap analysis also revealed high expression of DC markers in CD36$^+$ cells, accompanied by enriched MCC markers and low expression of other AE lineage markers (Figure 2G). Gene ontology (GO) analysis of the upregulated genes in CD36$^+$ cells showed enrichment in multiciliogenesis-related process (Figure 2H). These findings suggest that CD36$^+$ cells exhibit DC identity and are committed to the MCC lineage, with minimal contributions from other epithelial lineages. Functionally, CD36-based cell sorting followed by re-plating under ALI conditions showed that CD36$^+$ cells gave rise to MCC marker-positive cells, whereas CD36$^-$ cells exhibited limited MCC differentiation (Figures S2F and S2G). To assess the relevance of CD36 expression in native human AE, we reanalyzed public human lung scRNA-seq datasets, which showed little to no CD36 expression in DCs and MCCs of adult lungs and fetal lungs (Figure S2H). Consistently, IFA of adult and fetal lung tissues identified DC marker-positive cells without co-expression of CD36

Figure 1. Identification and characterization of DCs in human iAECs

(A) Schematic of iAEC generation via NKX2-1$^+$ hLP isolation, followed by 3D and subsequent ALI culture.

(B) UMAP of scRNA-seq of iAECs (201B7 iPSC line) on ALI day 20 following 3D culture.

(C) Violin plots of major AE lineage markers of MCC, SC, GC, BC, and DC.

(D and E) Feature plots of MCC (D) and DC (E) markers.

(F) Schematic of iAEC generation via direct seeding of CPM$^+$ hLPs onto ALI culture.

(G) Time-course IFA of iAECs (B2-3 iPSC line) by direct ALI differentiation on ALI days 0, 4, 8, 12, and 16 (after DAPT addition). FOXJ1 (red) and Ac-Tub (green) in row 1; CCNO (red) and NEK2 (white) in row 2; and FOXJ1 (red) and NEK2 (green) in row 3. Maximum intensity z-projections and orthogonal views are shown for each time point and marker combination. Nuclei, Hoechst 33342 (blue). Scale bars, 10 μm.

(H) Quantification of CCNO/NEK2- (top) and FOXJ1/NEK2-defined (bottom) cell populations during ALI differentiation (B2-3 iAECs) based on maximum intensity z-projections. Five randomly selected fields were quantified and averaged per differentiation. Stacked bar plots represent the mean percentage of each indicated cell population relative to the total number of nuclei from independent differentiations (n = 3). Numerical values (mean ± SEM) are provided in Table S1.

(I) Time-course RT-qPCR of *FOXJ1*, *SNTN*, and *CCNO* expression in iAECs (B2-3) on ALI days 0, 4, 8, 12, and 16. Data are presented as mean ± SEM (n = 4, independent experiments).

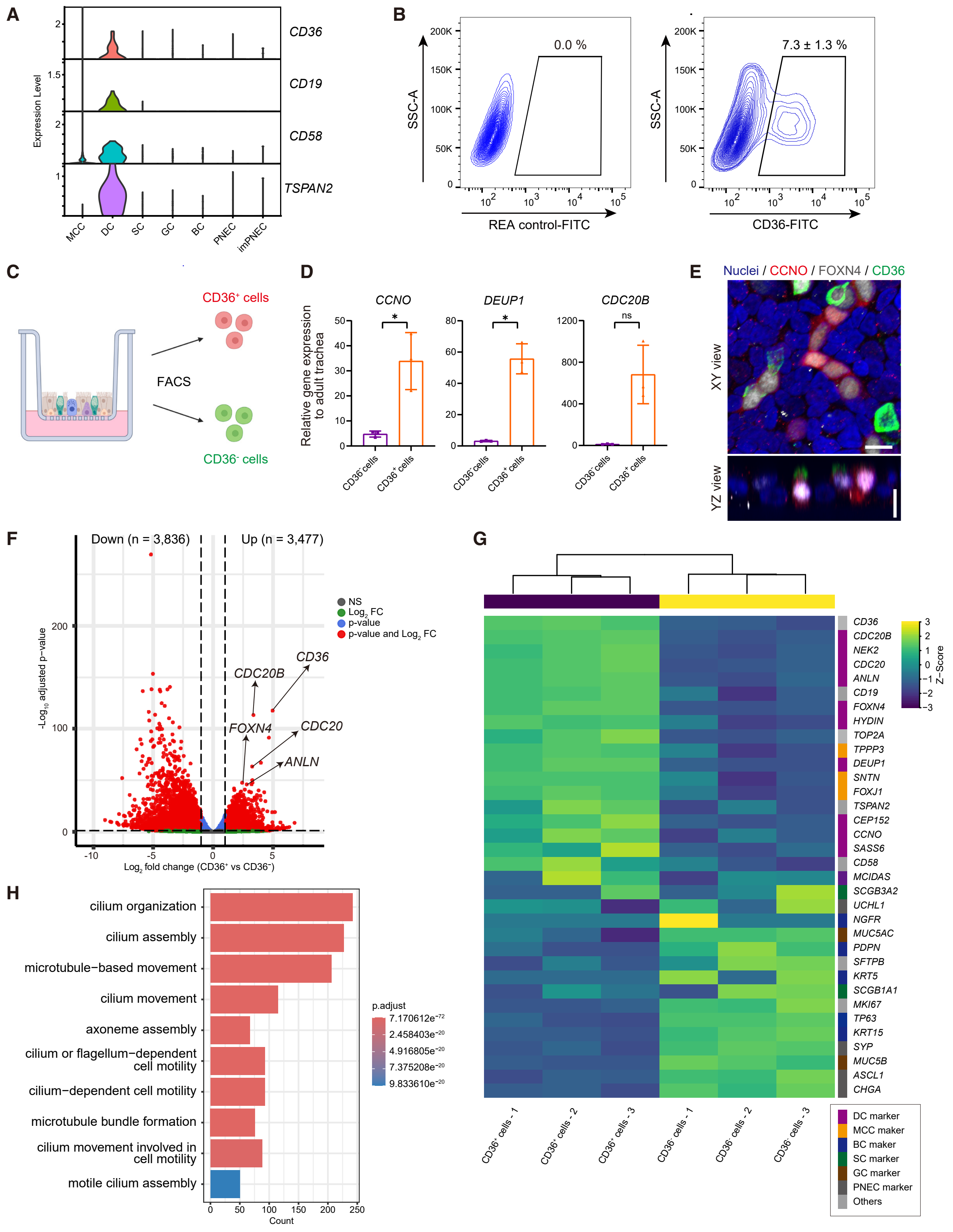

(legend on next page)

(Figure S2I). These results indicate that CD36 expression is not a prominent feature of DCs in native human AE, but rather emerges in iAECs, where it enables their isolation and functional analysis *in vitro*.

Generation and comparison between *CCNO*-variant and gene-corrected iPSCs

Next, we generated iPSCs from a 5-year-old patient with PCD harboring compound heterozygous *CCNO* variants: a nonsense variant in exon 1 (allele 1: c.262C>T; p.Gln88Ter) and a frameshift deletion in exon 3 (allele 2: c.781delC; p.Leu261fs) (Xu et al., 2024), hereafter termed as PCD-iPSCs, and established their isogenic control iPSCs by CRISPR/Cas9-mediated correction of the exon 1 variant for comparative analysis (Figures 3A and 3B), referred to as CCE1-iPSCs (*CCNO*-corrected exon 1). Both iPSC lines showed normal karyotypes (Figure S3A) and matched short tandem repeat (STR) profiles with the patient's peripheral blood mononuclear cells (PCD-PBMCs) (Figure S3B). The expression of pluripotency markers and trilineage differentiation were validated in PCD-iPSCs (Figure S3C). We differentiated both lines into CPM$^+$ hLPs, with no apparent difference in the induction efficiency of NKX2-1$^+$ cells (Figure S3D), and subsequently differentiated them into iAECs in ALI culture. *FOXJ1* and the motile cilia-specific marker *SNTN* were reduced in PCD-iAECs (Figure 3C). *CCNO* mRNA levels were not altered at most time points, except on day 4, possibly due to escape from nonsense-mediated decay by at least one of the variants. In contrast, the expression of several other DS-associated genes (Figure 3C) was decreased, suggesting impaired *CCNO* function. Additionally, no significant difference was observed in *NKX2-1*, a marker of hLPs, whereas the PNEC marker *SYP* was increased in PCD-iAECs. CCNO and acetylated tubulin (Ac-Tub) were decreased in PCD-iAECs, whereas FOXJ1 remained comparable between the two lines (Figure 3D). The number of CNTRL$^+$ puncta was decreased in PCD-iAECs (Figures 3E and 3F). Transmission electron microscopy (TEM) analysis showed that PCD-iAECs exhibited apical surfaces covered with microvilli but lacked recognizable basal bodies and cilia, indicating a defect in multiciliogenesis. In contrast, CCE1-iAECs had well-organized motile cilia with identifiable basal bodies anchored to the apical membrane (Figure 3G). CD36$^+$ cells were undetectable in PCD-iAECs, whereas CD36$^+$ cells with enriched DS-associated gene expression were detected in CCE1-iAECs (Figures 3H and S3E). As an independent validation, correction of the exon 3 variant in an isogenic iPSC line (CCE3-iPSCs; *CCNO*-corrected exon 3) showed a normal karyotype (Figure S3A) and STR profiles (Figure S3B). Consistently, CCE3-iAECs restored CCNO expression, thereby regaining the DC/MCC dynamics and multiciliogenesis, as confirmed by RT-qPCR and IFA (Figures S3F and S3G).

scRNA-seq reveals the role of CCNO in DC-mediated multiciliogenesis

We performed scRNA-seq comparative analysis of PCD- and CCE1-iAECs on day 8 after starting MCC induction, when DCs were enriched according to the RT-qPCR and IFA results (Figures 1G and 3C). UMAP of the integrated scRNA-seq datasets identified 12 distinct clusters and showed an increased representation of cells in intermediate AE differentiation states relative to the 3D-ALI-derived dataset (Figures 1B and 4A, 4C–4E, S4A, and S4B). DCs constituted 15.6% and 16.5% of total cells in PCD- and in CCE1-iAECs, respectively (Figure 4B). We identified two populations representing undifferentiated states. *CPM*-expressing cells with low levels of DC, MCC, and PNEC markers were defined as common progenitor cells (CPCs), reflecting an early stage of AE differentiation from CPM$^+$ hLPs. Cycling basal cells (cycBCs) were annotated based on the expression of BC- and proliferation-associated genes, as previously described (Plasschaert et al.,

Figure 2. CD36 enables the isolation of human iDCs

(A) Violin plots of candidate cell surface markers across clusters, with specific enrichment in the DC cluster, in iAECs (201B7) on ALI day 20 following 3D culture.

(B) Flow cytometry of CD36 in iAECs (B2-3) generated by direct ALI differentiation on ALI day 12. Data are presented as mean ± SEM ($n = 3$, independent experiments).

(C) Schematic of the flow cytometry-based strategy for sorting CD36$^+$ and CD36$^-$ cells from iAECs.

(D) RT-qPCR of DS-associated genes in CD36$^-$ and CD36$^+$ cells sorted from iAECs (B2-3) on ALI day 12. Data are presented as mean ± SEM ($n = 3$, independent experiments). $^*p < 0.05$; ns, not significant (paired t test).

(E) IFA of CD36 in iAECs (B2-3) on ALI day 8, with co-staining for CCNO and FOXN4. CD36 (green), CCNO (red), and FOXN4 (white) are shown. Maximum intensity z-projection (top) and orthogonal view (bottom). Hoechst (blue). Scale bars, 10 μm.

(F–H) Bulk RNA-seq analyses of CD36$^+$ and CD36$^-$ cells isolated from iAECs (B2-3) on ALI day 12 ($n = 3$, independent experiments). (F) Volcano plot for genes differentially expressed between CD36$^+$ and CD36$^-$ cells. Genes with $|\log_2$ fold change$| > 1$ and adjusted p value < 0.05 are considered significant (red). Selected DC marker genes are highlighted. (G) Heatmap of representative markers for major AE lineages in CD36$^+$ and CD36$^-$ cells. Gene expression values were scaled per row. Each column represents an individual sample replicate from the sorted populations. (H) GO analysis of biological process terms among differentially expressed genes between CD36$^+$ and CD36$^-$ cells. GO terms are ranked by adjusted p value, as indicated by the color. Bar length indicates the number of associated genes.

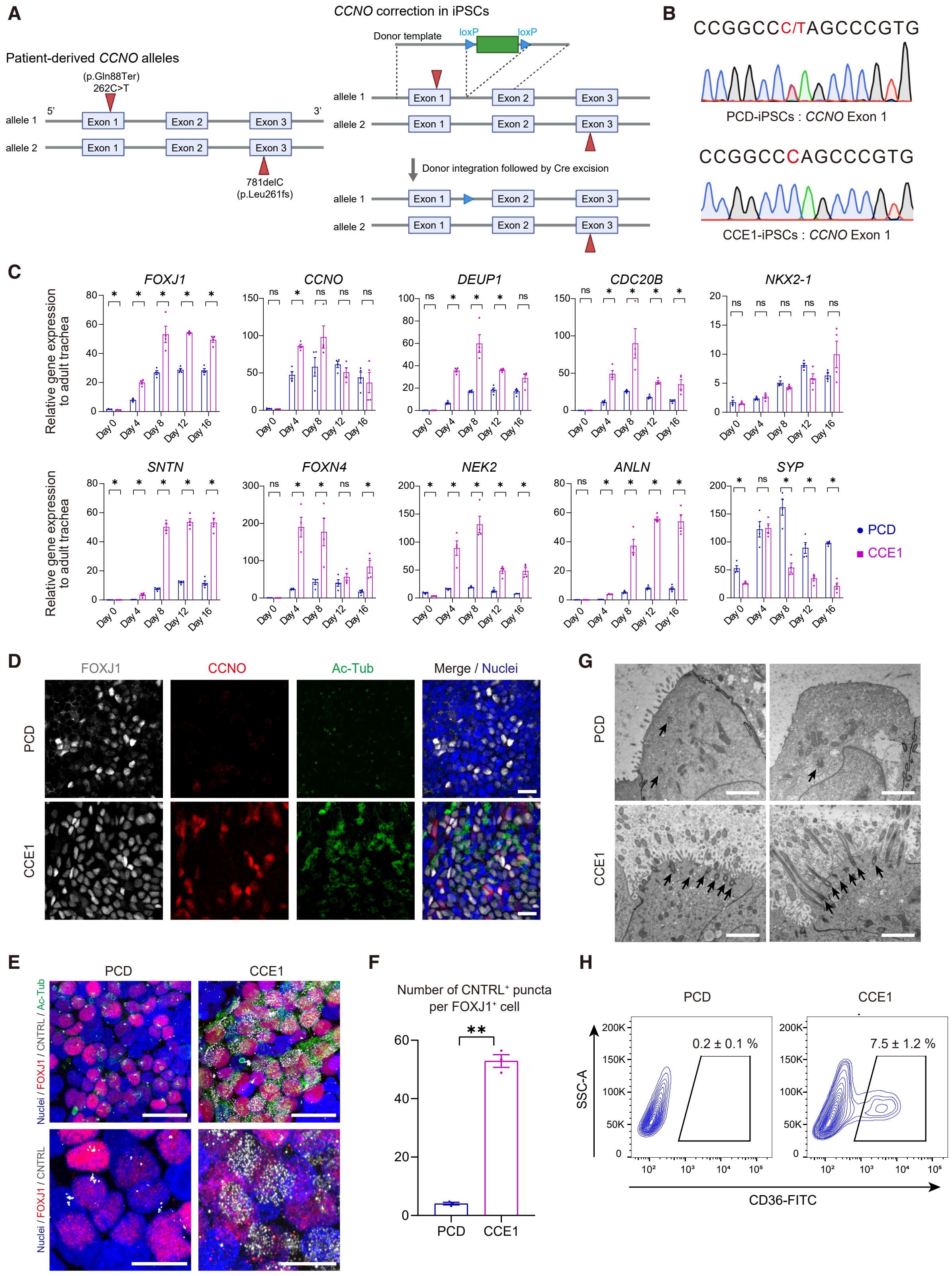

(legend on next page)

2018). DCs were further subdivided into DC1, DC2, and proliferative DCs (PDCs) subpopulations. DC1 was characterized by low *SNTN*, suggesting a less differentiated DC state. DC2 expressed *SNTN* and *TOP2A*, indicating a proliferating transitional stage between DC1 and MCCs. PDCs expressed proliferation- and DS-associated genes, suggesting a proliferative DC subset. In the DC2 cluster, *NEK2*, *ANLN*, and *CDC20* levels were lower in PCD-iAECs than in CCE1-iAECs, implying that these genes may act downstream of *CCNO* to regulate DC maturation. The two clusters expanded more in PCD-iAECs than in CCE1-iAECs. One cluster lacked the expression of DS-associated genes and *SNTN* and was defined as non-deuterosomal precursor MCCs (nd-preMCCs), while the other expressed *FOXJ1* but exhibited low *SNTN* with minimal expression of DS-associated genes and structural genes for motile cilia and was defined as aberrant MCCs (abMCCs). The proportion of MCCs in PCD-iAECs decreased (15.6%) compared with CCE1-iAECs (45.9%), indicating that the expansion of nd-preMCCs and abMCCs was associated with defective MCC differentiation in PCD-iAECs. In addition, PNEC populations modestly increased in PCD-iAECs (Figure 4B). To assess the developmental maturity of these AE lineages, we compared module scores of DCs, MCCs, and PNECs between iAECs and adult and fetal human lung scRNA-seq datasets (Figures S4C and S4D). DC2 cells derived from CCE1-iPSCs showed module scores comparable to adult lung DCs, whereas those from PCD-iPSCs were closer to fetal lung DCs. MCCs derived from CCE1-iPSCs exhibited module scores comparable to both fetal and adult MCCs, while those derived from PCD-iPSCs showed a slightly broader score distribution. The iPSC-derived PNECs were more closely aligned with fetal than adult PNECs. In CCE1-iAECs, pseudotime analysis revealed two differentiation trajectories: one proceeding from CPCs through DC1 and DC2 to MCCs and another branching from CPCs to PDCs (Figure 4G). Among these, the CPC-DC1-DC2-MCC pathway was predominant, consistent with progressive upregulation of MCC-associated genes (Figure S4E). In contrast, PCD-iAECs displayed an additional trajectory from CPCs to MCCs via nd-preMCCs, bypassing the DC states (Figures 4F and S4F). Integration of the two datasets supported this shift: the proportion of cells following the CPC-DC1-DC2-MCC trajectory was reduced in PCD-iAECs, whereas more cells followed the CPC-nd-preMCC-MCC trajectory. RNA velocity analysis independently confirmed these findings. CCE1-iAECs exhibited directional flow from DC1 to DC2 to MCCs, with bidirectional vectors between DC1 and DC2, consistent with a transient deuterosomal state. This dynamic was largely absent in PCD-iAECs, which showed reduced DC-associated flux and increased DC-bypassing trajectories with emergence of nd-preMCCs toward MCCs (Figure S4G). In IFA, the cells co-expressing CDC20B, NEK2, and ANLN in CCE1-iAECs were consistent with the transcriptional profile of DC2 (Figure 4H). In contrast, NEK2 and ANLN in PCD-iAECs were mainly expressed in $CDC20B^{-}$ cells, suggesting a shift from a normal DC2 identity to an aberrant DC2-like phenotype, along with a relative increase in PDCs. The proportions of $NEK2^{+}$ and $ANLN^{+}$ cells among $CDC20B^{+}$ cells were markedly reduced in PCD-iAECs compared with CCE1-iAECs (NEK2: 2.16 ± 0.39% vs. 39.1 ± 8.9%; ANLN: 1.60 ± 0.37% vs. 24.5 ± 1.8%) (Figure 4I). These results suggest that CCNO dysfunction disrupts DC-mediated multiciliogenesis and promotes an aberrant DC-bypassing differentiation.

DISCUSSION

This study identified DCs in human iAECs. To date, studies on the DS pathway and DCs have predominantly

Figure 3. Generation and analysis of iPSCs from a patient with PCD harboring *CCNO* variants and their gene-corrected iPSCs
(A) Schematic of compound heterozygous *CCNO* variants identified in the patient with PCD (left), and the gene editing strategy used to generate isogenic CCE1-iPSCs (right).
(B) Sanger sequencing of the *CCNO* exon 1 region in PCD- and CCE1-iPSCs, showing successful correction of the nonsense variant. The heterozygous C>T variant in PCD-iPSCs and the corrected wild-type C in CCE1-iPSCs are indicated in red.
(C) Time-course RT-qPCR of DS-associated and selected AE lineage markers (MCCs, hLPs, and PNECs) during iAEC differentiation in PCD- and CCE1-iAECs (n = 4 replicates at each time point). Data are presented as mean ± SEM. $*p < 0.05$ (Mann-Whitney U test).
(D) Maximum intensity z-projections of IFA in PCD- and CCE1-iAECs on ALI day 8. FOXJ1 (white), CCNO (red), and Ac-Tub (green) are shown. Hoechst (blue). Scale bars, 20 μm.
(E) IFA for CNTRL in PCD- and CCE1-iAECs on ALI day 16. Maximum intensity z-projections at low (top images) and high (bottom images) magnification. Top images show FOXJ1 (red), CNTRL (white), and Ac-Tub (green); bottom images show FOXJ1 (red) and CNTRL (white). Hoechst (blue). Scale bars, 20 μm (top images); 10 μm (bottom images).
(F) Quantification of $CNTRL^{+}$ puncta per $FOXJ1^{+}$ cell based on maximum intensity z-projections. Five randomly selected fields were analyzed per differentiation. Data are presented as mean ± SEM (n = 3, independent differentiations). $**p < 0.01$ (paired t test).
(G) TEM of PCD- and CCE1-iAECs on day 41 of 3D culture. Arrows indicate basal bodies. Scale bars, 2 μm.
(H) Flow cytometry of $CD36^{+}$ cell proportions in PCD- and CCE1-iAECs on ALI days 4–16 (n = 3 for PCD-iAECs; n = 6 for CCE1-iAECs). Data are presented as mean ± SEM.

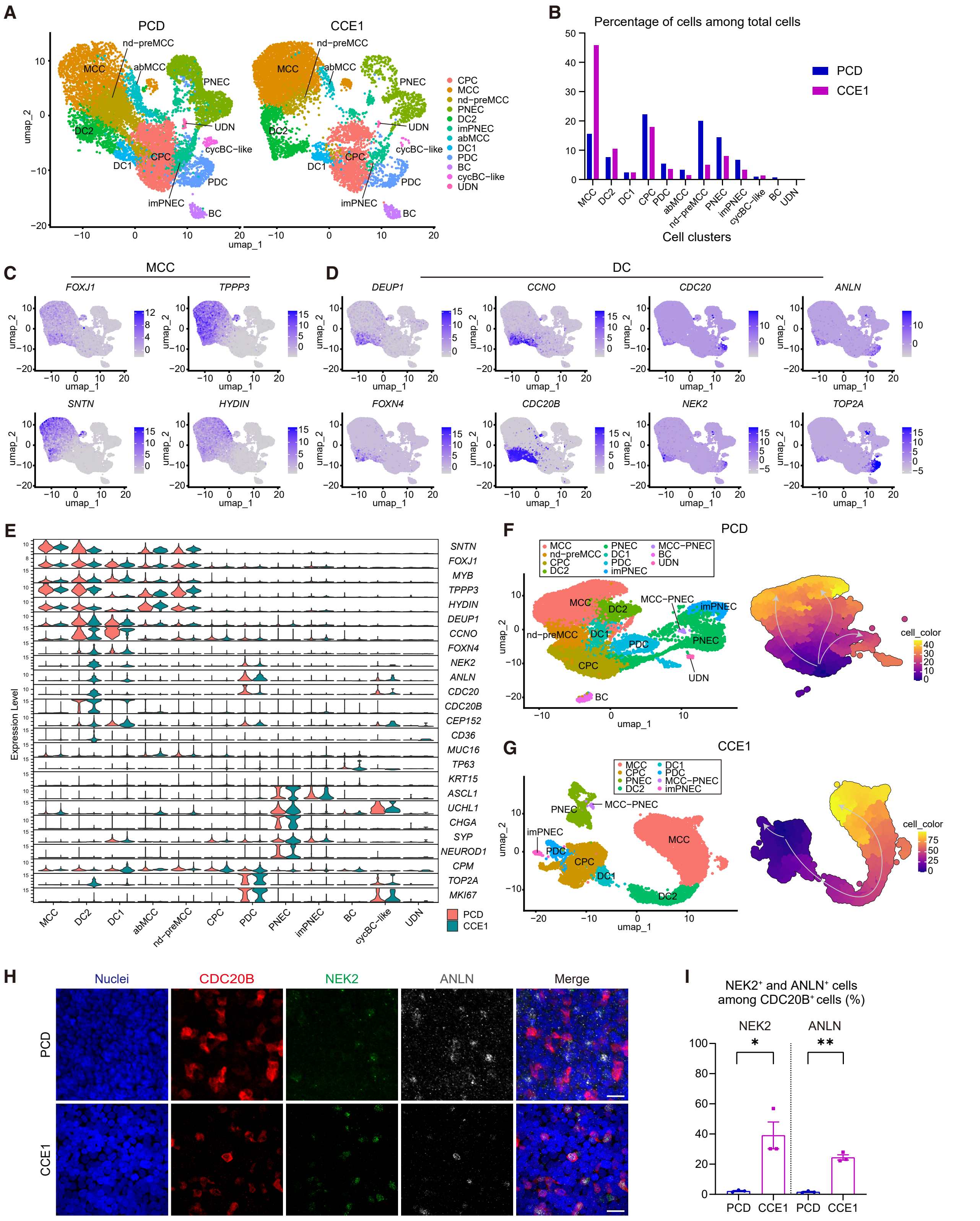

(legend on next page)

been conducted in Xenopus (Zhao et al., 2013; Wallmeier et al., 2014; Revinski et al., 2018) and mouse models (Mercey et al., 2019). Human studies on DC biology remain limited (Revinski et al., 2018; Ruiz García et al., 2019). Our human iPSC-based system provides stable access to DCs, enabling analysis of regulatory transitions during human multiciliogenesis. Integration of our scRNA-seq data with the public adult and fetal human lung atlases enabled a quantitative assessment of model fidelity and developmental maturity (Figure S4C). DC2 cells derived from CCE1-iPSCs closely resembled adult human lung DCs, whereas MCCs derived from CCE1-iPSCs exhibited almost equivalent module scores to fetal and adult MCCs. In contrast, MCCs derived from PCD-iPSCs showed a slightly broader score distribution, suggesting that multiciliogenesis is impaired due to CCNO deficiency. Together, these findings demonstrate that the iAECs capture lineage-specific transcriptional programs while revealing disease-associated delays in DC maturation and MCC differentiation. We isolated DCs from iAECs using CD36 as a surface antigen, which would facilitate downstream applications to study multiciliogenesis *in vitro*. While non-human models have linked CCNO, MCIDAS, and DEUP1 to DS regulation (Ma et al., 2014; Zhao et al., 2013; Funk et al., 2015), its regulation in humans remains unclear. We identified *ANLN*, *NEK2*, and *CDC20* as CCNO-downregulated genes in DC2 of PCD-iAECs, suggesting a transcriptional axis required for DS-mediated centriole amplification, consistent with recent findings implicating CCNO in a noncanonical cell-cycle program during MCC differentiation (Khoury Damaa et al., 2025). The difference between transcript- and protein-level measurements for DS-associated genes reflects cell-state-specific regulation during DS maturation. Reduced *NEK2* and *ANLN* expression was confined to the late DC2 state in PCD-iAECs, whereas IFA did not reveal a global reduction in protein abundance across the entire epithelial population. In the present study, PCD-iAECs with *CCNO* variants showed an increase in PDCs co-expressing proliferation- and DS-related genes, suggesting an impaired exit from the proliferative state and failure to complete differentiation. We speculate that this aberrant pathway reflects compensatory use of the mother centriole-dependent pathway, which may be insufficient for full MCC maturation. In addition to impaired DC-mediated multiciliogenesis, CCNO deficiency altered AE lineage balance within this *in vitro* system, including a modest increase in PNEC populations. Given that *CCNO* was not expressed in immature PNECs (imPNECs), this change likely reflects an indirect consequence of defective MCC differentiation. Together, these findings indicate that, within the context of CCNO-associated PCD, disruption of the DS-dependent DC-to-MCC differentiation pathway underlies the primary defect, whereas changes in other epithelial lineages arise as secondary effects of impaired multiciliogenesis.

METHODS

Ethics

The generation and use of patient-derived iPSCs and the use of human lung tissue were approved by the Ethics Committee of the Graduate School and Faculty of Medicine and CiRA, Kyoto University (R91/G259 and R1009/G1074). Written informed consent was obtained from the patients' legal guardians.

Statistical analysis

Statistical analyses of the data were performed using GraphPad Prism version 8 (GraphPad Software) and R. Data are presented as mean ± standard error of the mean (SEM). Comparisons between the two groups were conducted using the Mann-Whitney *U* test or paired *t* test, as appropriate. Statistical significance was set at $p < 0.05$. The statistical tests used are described in the figure legends.

Figure 4. scRNA-seq comparative analysis between PCD- and CCE1-iAECs reveals CCNO-dependent impairment of DC-mediated multiciliogenesis

(A) UMAP of the integrated scRNA-seq, shown separately for PCD-iAECs (left) and CCE1-iAECs (right), on ALI day 8. imPNEC, immature PNEC; UDN, undetermined.

(B) Proportion of each cluster among total cells in PCD- and CCE1-iAECs in the scRNA-seq from ALI day 8 cultures.

(C and D) Feature plots of representative MCC (C) and DC (D) markers.

(E) Violin plots of major AE and DC markers in PCD- and CCE1-iAECs.

(F and G) Left: UMAP of pre-integration scRNA-seq derived from PCD-iAECs (F) and CCE1-iAECs (G). Right: Visualization of MCC/DC-related clusters in each condition along the pseudotime trajectory based on pre-integration data, color-coded by pseudotime values.

(H) IFA for CDC20B (red), NEK2 (green), and ANLN (white) in PCD- and CCE1-iAECs on ALI day 8. Maximum intensity z-projections are shown. Hoechst (blue). Scale bars, 20 μm.

(I) Quantification of $NEK2^+$ (left) and $ANLN^+$ (right) cells among $CDC20B^+$ cells in PCD- and CCE1-iAECs. Five randomly selected fields were analyzed per condition. Data are presented as mean ± SEM ($n = 3$, independent differentiations). $^*p < 0.05$; $^{**}p < 0.01$ (paired t test).

RESOURCE AVAILABILITY

Lead contact

Requests for further information and resources should be directed to and will be fulfilled by the lead contact, Shimpei Gotoh (gotoh.shimpei.5m@kyoto-u.ac.jp).

Materials availability

Materials used in this study are available upon request under a completed material transfer agreement.

Data and code availability

Bulk and single-cell RNA-seq data generated in this study have been deposited in the Gene Expression Omnibus (GEO: GSE299246 and GSE299245), and in the DNA DataBank of Japan (DDBJ: J-DS000923). The data will be made publicly available upon publication.

ACKNOWLEDGMENTS

We thank S. Sakurai and K. Deguchi of the genome analysis group in the CiRA Common Equipment Management Office for RNA-seq library preparation and sequencing with analysis; S. Ikeo and Y. Yamamoto for supporting the generation of anti-CPM antibody in the past; and K. Okamoto-Furuta and T. Katsuno at the Division of Electron Microscopic Study, Center for Anatomical Studies, Kyoto University, for supporting electron microscopy. This study was funded by JSPS KAKENHI (JP22H03077, JP23K24338, JP25K12809, and JP25K02660); AMED (JP19ek0109410, JP23bm1323001, and JP25bk0104190); the Fujiwara Memorial Foundation; the Naito Foundation; and the iPS Cell Research Fund for CiRA at Kyoto University. Schematic illustrations in this manuscript were created using BioRender.com.

AUTHOR CONTRIBUTIONS

Conceptualization, H.Y., S.K., and S.G.; methodology, H.Y., S.K., Ko.T., N.S., S.T., and S.G.; validation, S.K. and N.S.; formal analysis, H.Y., S.K., T.Y., and S.G.; investigation, H.Y., S.K., Ko.T., S.T., and S.G.; resources, Y.X., T.T., H.O., Ka.T., and S.G.; writing, H.Y., S.K., and S.G.; supervision, T.H. and Ka.T.

DECLARATION OF INTERESTS

S.G. is the founder and shareholder of HiLung, Inc. S.K. and S.G. are the inventors of Kyoto University's patents related to the method of generating AE: WO2016148307A1.

SUPPLEMENTAL INFORMATION

Supplemental information can be found online at https://doi.org/10.1016/j.stemcr.2026.102860.

Received: June 20, 2025
Revised: February 19, 2026
Accepted: February 20, 2026
Published: March 19, 2026

REFERENCES

Al Jord, A., Lemaître, A.-I., Delgehyr, N., Faucourt, M., Spassky, N., and Meunier, A. (2014). Centriole amplification by mother and daughter centrioles differs in multiciliated cells. Nature *516*, 104–107.

Boon, M., Wallmeier, J., Ma, L., Loges, N.T., Jaspers, M., Olbrich, H., Dougherty, G.W., Raidt, J., Werner, C., Amirav, I., et al. (2014). MCIDAS mutations result in a mucociliary clearance disorder with reduced generation of multiple motile cilia. Nat. Commun. *5*, 4418.

Brody, S.L., Pan, J., Huang, T., Xu, J., Xu, H., Koenitizer, J.R., Brennan, S.K., Nanjundappa, R., Saba, T.G., Rumman, N., et al. (2025). Undocking of an extensive ciliary network induces proteostasis and cell fate switching resulting in severe primary ciliary dyskinesia. Sci. Transl. Med. *17*, eadp5173.

Khoury Damaa, M., Serizay, J., Balagué, R., Boudjema, A.-R., Faucourt, M., Delgehyr, N., Goh, K.J., Lu, H., Tan, E.K., James, C.T., et al. (2025). Cyclin O controls entry into the cell-cycle variant required for multiciliated cell differentiation. Cell Rep. *44*, 115117.

Funk, M.C., Bera, A.N., Menchen, T., Kuales, G., Thriene, K., Lienkamp, S.S., Dengjel, J., Omran, H., Frank, M., and Arnold, S.J. (2015). Cyclin O (Ccno) functions during deuterosome-mediated centriole amplification of multiciliated cells. EMBO J. *34*, 1078–1089.

Ruiz García, S., Deprez, M., Lebrigand, K., Cavard, A., Paquet, A., Arguel, M.-J., Magnone, V., Truchi, M., Caballero, I., Leroy, S., et al. (2019). Novel dynamics of human mucociliary differentiation revealed by single-cell RNA sequencing of nasal epithelial cultures. Development *146*, dev177428.

Gotoh, S., Ito, I., Nagasaki, T., Yamamoto, Y., Konishi, S., Korogi, Y., Matsumoto, H., Muro, S., Hirai, T., Funato, M., et al. (2014). Generation of alveolar epithelial spheroids via isolated progenitor cells from human pluripotent stem cells. Stem Cell Rep. *3*, 394–403.

Hannah, W.B., Seifert, B.A., Truty, R., Zariwala, M.A., Ameel, K., Zhao, Y., Nykamp, K., and Gaston, B. (2022). The global prevalence and ethnic heterogeneity of primary ciliary dyskinesia gene variants: a genetic database analysis. Lancet Respir. Med. *10*, 459–468.

Hawkins, F.J., Suzuki, S., Beermann, M.L., Barillà, C., Wang, R., Villacorta-Martin, C., Berical, A., Jean, J.C., Le Suer, J., Matte, T., et al. (2021). Derivation of airway basal stem cells from human pluripotent stem cells. Cell Stem Cell *28*, 79–95.e8.

He, P., Lim, K., Sun, D., Pett, J.P., Jeng, Q., Polanski, K., Dong, Z., Bolt, L., Richardson, L., Mamanova, L., et al. (2022). A human fetal lung cell atlas uncovers proximal-distal gradients of differentiation and key regulators of epithelial fates. Cell *185*, 4841–4860.e25.

Konishi, S., Gotoh, S., Tateishi, K., Yamamoto, Y., Korogi, Y., Nagasaki, T., Matsumoto, H., Muro, S., Hirai, T., Ito, I., et al. (2016). Directed induction of functional multiciliated cells in proximal airway epithelial spheroids from human pluripotent stem cells. Stem Cell Rep. *6*, 18–25.

Ma, L., Quigley, I., Omran, H., and Kintner, C. (2014). Multicilin drives centriole biogenesis via E2f proteins. Genes Dev. *28*, 1461–1471.

Mercey, O., Levine, M.S., LoMastro, G.M., Rostaing, P., Brotslaw, E., Gomez, V., Kumar, A., Spassky, N., Mitchell, B.J., Meunier, A., et al.

(2019). Massive centriole production can occur in the absence of deuterosomes in multiciliated cells. Nat. Cell Biol. *21*, 1544–1552.

Plasschaert, L.W., Žilionis, R., Choo-Wing, R., Savova, V., Knehr, J., Roma, G., Klein, A.M., and Jaffe, A.B. (2018). A single-cell atlas of the airway epithelium reveals the CFTR-rich pulmonary ionocyte. Nature *560*, 377–381.

Revinski, D.R., Zaragosi, L.-E., Boutin, C., Ruiz-Garcia, S., Deprez, M., Thomé, V., Rosnet, O., Gay, A.-S., Mercey, O., Paquet, A., et al. (2018). CDC20B is required for deuterosome-mediated centriole production in multiciliated cells. Nat. Commun. *9*, 4668.

Sone, N., Konishi, S., Igura, K., Tamai, K., Ikeo, S., Korogi, Y., Kanagaki, S., Namba, T., Yamamoto, Y., Xu, Y., et al. (2021). Multicellular modeling of ciliopathy by combining iPS cells and microfluidic airway-on-a-chip technology. Sci. Transl. Med. *13*, eabb1298.

Wallmeier, J., Al-Mutairi, D.A., Chen, C.-T., Loges, N.T., Pennekamp, P., Menchen, T., Ma, L., Shamseldin, H.E., Olbrich, H., Dougherty, G.W., et al. (2014). Mutations in CCNO result in congenital mucociliary clearance disorder with reduced generation of multiple motile cilia. Nat. Genet. *46*, 646–651.

Xu, Y., Ueda, K., Nishikido, T., Matsumoto, T., and Takeuchi, K. (2024). Two Japanese pediatric patients with primary ciliary dyskinesia caused by loss-of-function variants in the CCNO gene. Cureus *16*, e58854.

Zhao, H., Zhu, L., Zhu, Y., Cao, J., Li, S., Huang, Q., Xu, T., Huang, X., Yan, X., and Zhu, X. (2013). The Cep63 paralogue Deup1 enables massive de novo centriole biogenesis for vertebrate multiciliogenesis. Nat. Cell Biol. *15*, 1434–1444.

Stem Cell Reports, Volume *21*

Supplemental Information

Deuterosomal cells are the responsible lineage for multiciliogenesis in human airway differentiation

Haruka Yamaki, Satoshi Konishi, Koji Tamai, Naoyuki Sone, Senye Takahashi, Yifei Xu, Takahiro Tsuji, Hiroaki Ozasa, Takuya Yamamoto, Toyohiro Hirai, Kazuhiko Takeuchi, and Shimpei Gotoh

SUPPLEMENTAL INFORMATION

Supplemental Figures

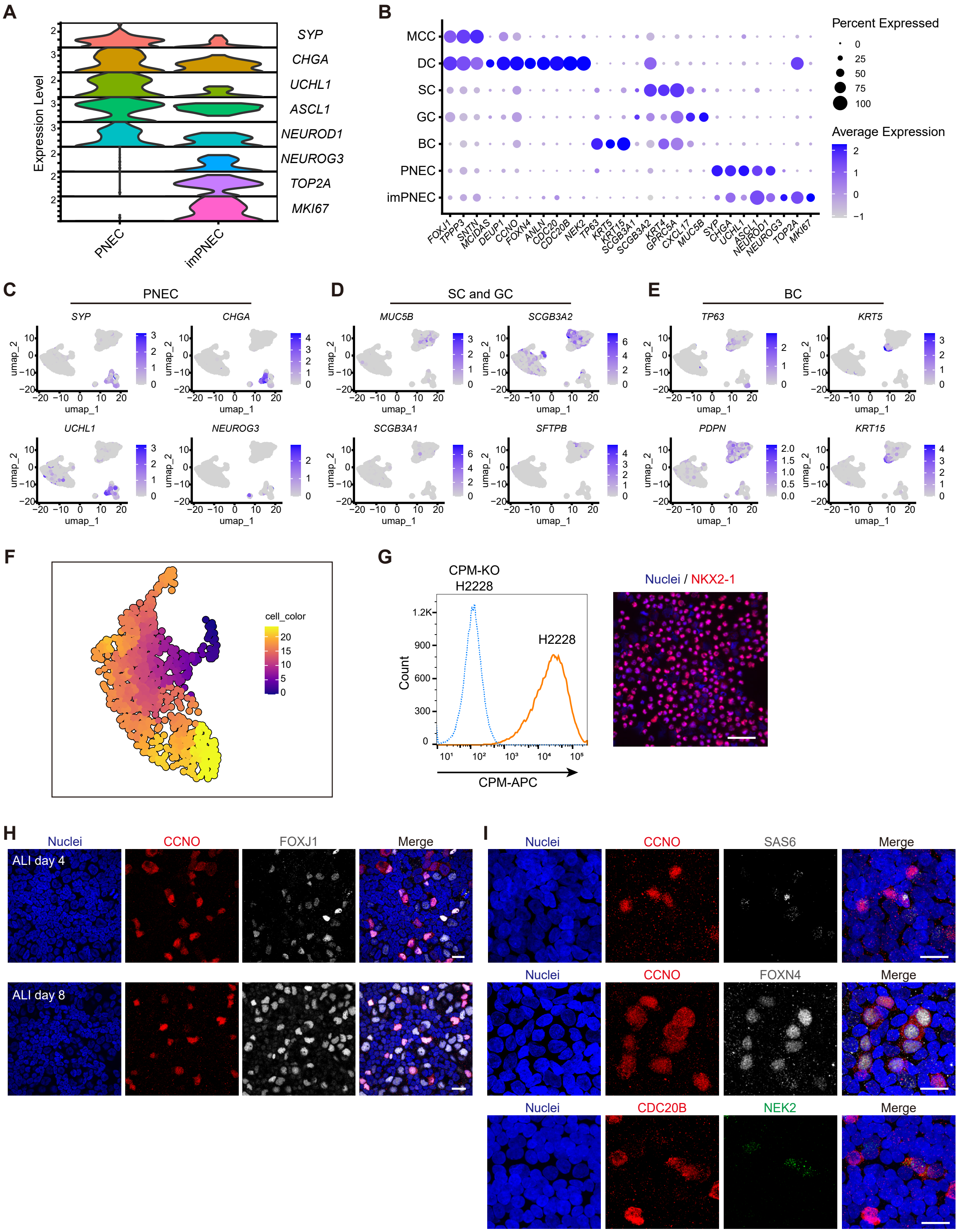

Figure S1. Extended characterization of iAEC subpopulations and validation of DC markers in ALI culture, related to Figure 1.

(A) Violin plots show the expression of canonical pulmonary neuroendocrine cell (PNEC) markers and proliferation markers in clusters corresponding to PNEC lineage in iAECs (201B7 iPSC line) on ALI day 20 following 3D culture.

(B) Dot plot shows expression and detection rates of representative markers among all identified AE clusters.

(C-E) Feature plots of representative markers for PNEC (C), SC and GC (D), and BC (E).

(F) Pseudotime analysis of DCs and MCCs shows differentiation trajectory from DCs to mature MCCs.

(G) Validation of rat monoclonal anti-CPM antibody. Left: Flow cytometry analysis of CPM staining in H2228 cells (orange) that endogenously express CPM, and in CPM-knockout H2228 cells (blue), confirming antibody specificity. Right: IFA for NKX2-1 in CPM^+ sorted cells on day 14 of hLP differentiation from B2-3 iPSC line. Nuclei were counterstained with Hoechst 33342 (blue). Scale bars, 50 μm.

(H) IFA for CCNO (red) and FOXJ1 (white) in iAECs (B2-3) under direct ALI differentiation condition on days 4 and 8. Single optical sections are shown. Hoechst (blue). Scale bars, 20 μm.

(I) IFA for DS-associated markers in iAECs (B2-3) cultured under ALI on day 8 shows CCNO (red) and SAS6 (white) in row 1, CCNO (red) and FOXN4 (white) in row 2, and CDC20B (red) and NEK2 (green) in row 3. Maximum intensity z-projections are shown. Hoechst (blue). Scale bars, 20 μm.

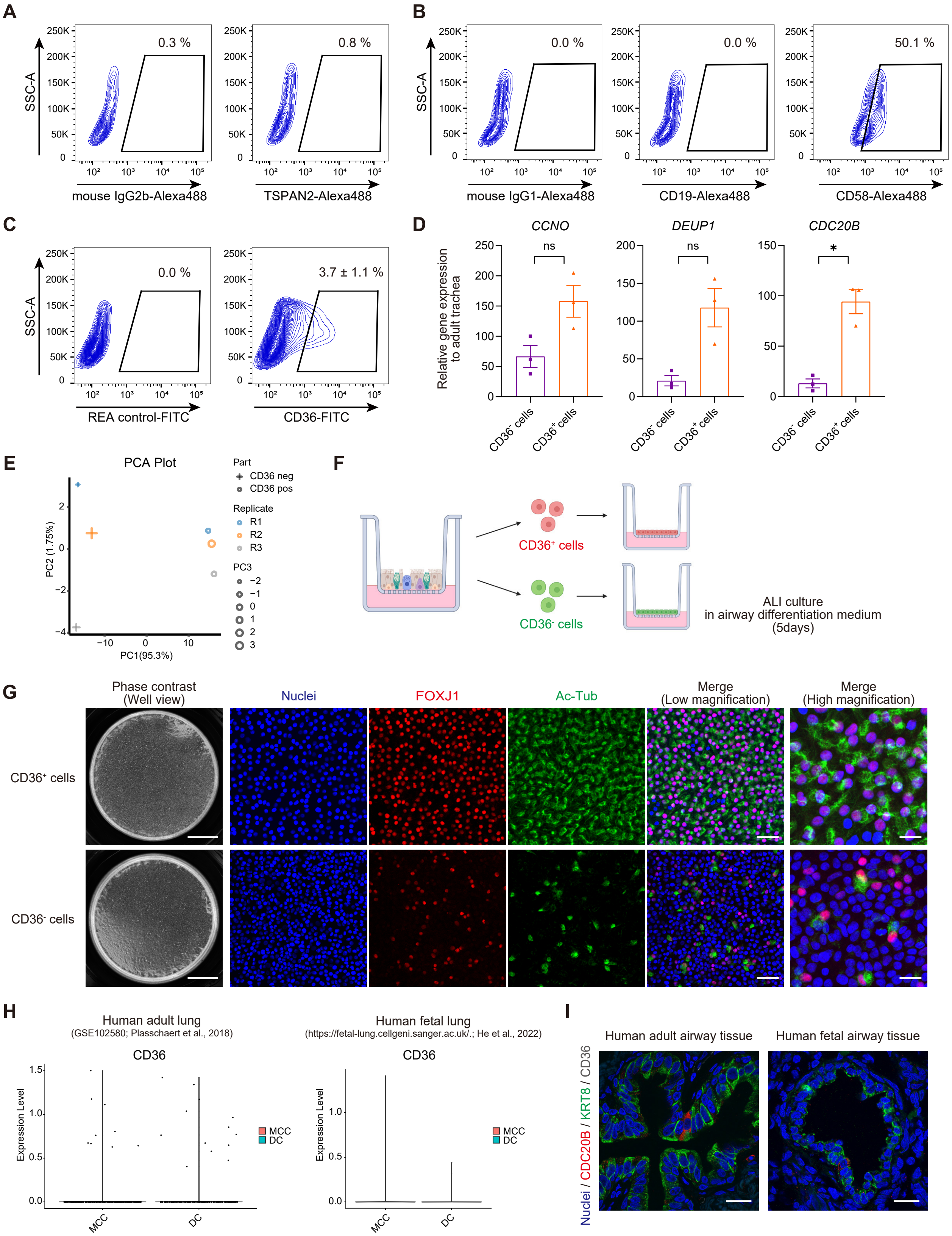

Figure S2. Evaluation of candidate surface markers for DCs and validation of CD36-based cell sorting, related to Figure 2.

(A, B) Flow cytometry of candidate cell surface markers other than CD36 in iAECs in ALI culture. (A) TSPAN2 analyzed in day 12 iAECs derived from B2-3 iPSC line. (B) CD19 and CD56 analyzed in day 16 iAECs derived from CCE1 (*CCNO*-corrected exon 1) iPSC line.

(C) Flow cytometry of CD36 in iAECs derived from the 585A1 iPSC line on ALI days 4, 8, and 12. Data from three independent time points (n = 1 each) were combined and are presented as mean ± SEM (n = 3).

(D) RT-qPCR of DS-associated genes in $CD36^-$ and $CD36^+$ cells sorted from iAECs derived from the healthy donor-derived 585A1 iPSC line. Cells were collected on ALI days 4, 8, 12 (n = 1 per time point). Data from three time points were combined and are presented as mean ± SEM (n = 3 per group). * $P < 0.05$ (paired *t*-test).

(E) PCA of gene expression profiles in $CD36^+$ and $CD36^-$ cells isolated from iAECs (B2-3) on ALI day 12 (n = 3, independent experiments). PCA was performed on the top 300 most variable genes (log-transformed TPM values) across all samples. Differences in gene expression between groups were assessed using the Wald test implemented in DESeq2, and adjusted *P*-values were calculated using the Benjamini–Hochberg method.

(F) Schematic overview of CD36-based cell sorting and subsequent re-plating for AE differentiation.

(G) Representative images of AE cultures on day 5 after CD36-based sorting and re-plating. The leftmost column shows phase-contrast images of the entire well. The right columns show IFA of iAECs (B2-3) derived from $CD36^−$ (top row) and $CD36^+$ (bottom row) populations. Cells were stained for FOXJ1 (red), and Ac-Tub (green). Hoechst (blue). Scale bars, 1000 μm (phase contrast); 50 μm (low magnification IFA); and 20 μm (high magnification IFA).

(H) Violin plots show CD36 expression in MCCs and DCs based on reanalysis of public human lung scRNA-seq datasets. Adult human lung data (left) were obtained from GSE102580 (Plasschaert et al., 2018). Fetal human lung data (right) were obtained from https://fetal-lung.cellgeni.sanger.ac.uk/ (He et al., 2022).

(I) IFA of human airway tissue sections. Adult (left) and fetal (right) airway tissues were stained for CDC20B (red), KRT8 (green; epithelial cell marker), and CD36 (white). Hoechst (blue). Scale bars, 20 μm.

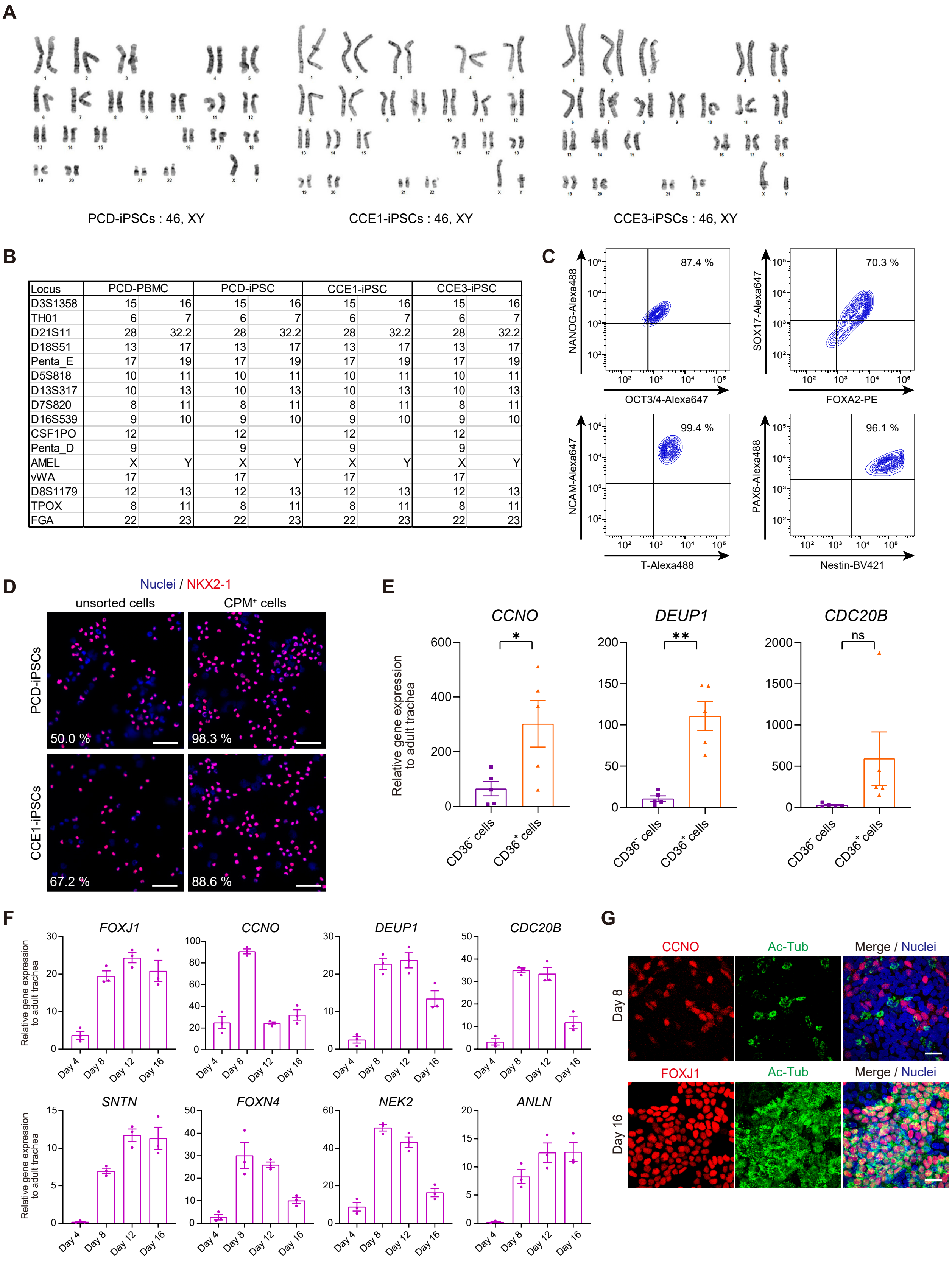

Locus	PCD-PBMC		PCD-iPSC		CCE1-iPSC		CCE3-iPSC	
D3S1358	15	16	15	16	15	16	15	16
TH01	6	7	6	7	6	7	6	7
D21S11	28	32.2	28	32.2	28	32.2	28	32.2
D18S51	13	17	13	17	13	17	13	17
Penta_E	17	19	17	19	17	19	17	19
D5S818	10	11	10	11	10	11	10	11
D13S317	10	13	10	13	10	13	10	13
D7S820	8	11	8	11	8	11	8	11
D16S539	9	10	9	10	9	10	9	10
CSF1PO	12		12		12		12	
Penta_D	9		9		9		9	
AMEL	X	Y	X	Y	X	Y	X	Y
vWA	17		17		17		17	
D8S1179	12	13	12	13	12	13	12	13
TPOX	8	11	8	11	8	11	8	11
FGA	22	23	22	23	22	23	22	23

Figure S3. Validation of PCD-, CCE1-, and CCE3-iPSC lines, and CD36-based enrichment of iDCs, related to Figure 3.

(A) Chromosomal analysis of PCD-iPSCs (clone CiRA01103-11), CCE1-iPSCs (clone CCE1 71-16) and CCE3-iPSCs (clone CCE3 42-2), reveals normal karyotypes in all cell lines.

(B) STR analysis of PCD-PBMCs and PCD-, CCE1-, and CCE3-iPSCs shows identical short tandem repeat patterns in all samples.

(C) Flow cytometry of undifferentiated markers (OCT3/4 and NANOG) in PCD-iPSCs (top left), followed by endoderm (FOXA2 and SOX17; top right), mesoderm (T and NCAM; bottom left), and ectoderm (Nestin and PAX6; bottom right) markers in PCD-iPSCs after trilineage differentiation.

(D) IFA of NKX2-1 in PCD- and CCE1-iPSCs on day 21 of hLP differentiation shows the ratios of NKX2-1$^+$ cells among all nuclei. Left: unsorted cells; right: CPM$^+$ cells sorted by MACS. Hoechst (blue). Scale bars, 50 μm.

(E) RT-qPCR of DS-associated genes in CD36$^-$ and CD36$^+$ cells sorted from iAECs derived from CCE1-iPSCs. Cells were collected on ALI days 4, 8, 11, and 16, with two replicates for day 8 (n = 5 independent samples). Paired *t*-tests were used for *CCNO* and *DEUP1*. Shapiro–Wilk test results indicated non-normal distribution of *CDC20B*, which was assessed using Wilcoxon signed-rank test. Data are shown as mean ± SEM (n = 5 per group). * $P < 0.05$; ** $P < 0.01$.

(F) Time-course RT-qPCR of DS-associated and MCC markers during iAEC differentiation in CCE3-iAECs (n = 3 replicates per time point).

(G) Maximum intensity z-projections of IFA in CCE3-iAECs on ALI days 8 and 16. Top row shows cells on ALI day 8 stained for CCNO (red) and Ac-Tub (green). Bottom row shows cells on ALI day 16 stained for FOXJ1 (red) and Ac-Tub (green). Hoechst (blue). Scale bars, 20 μm.

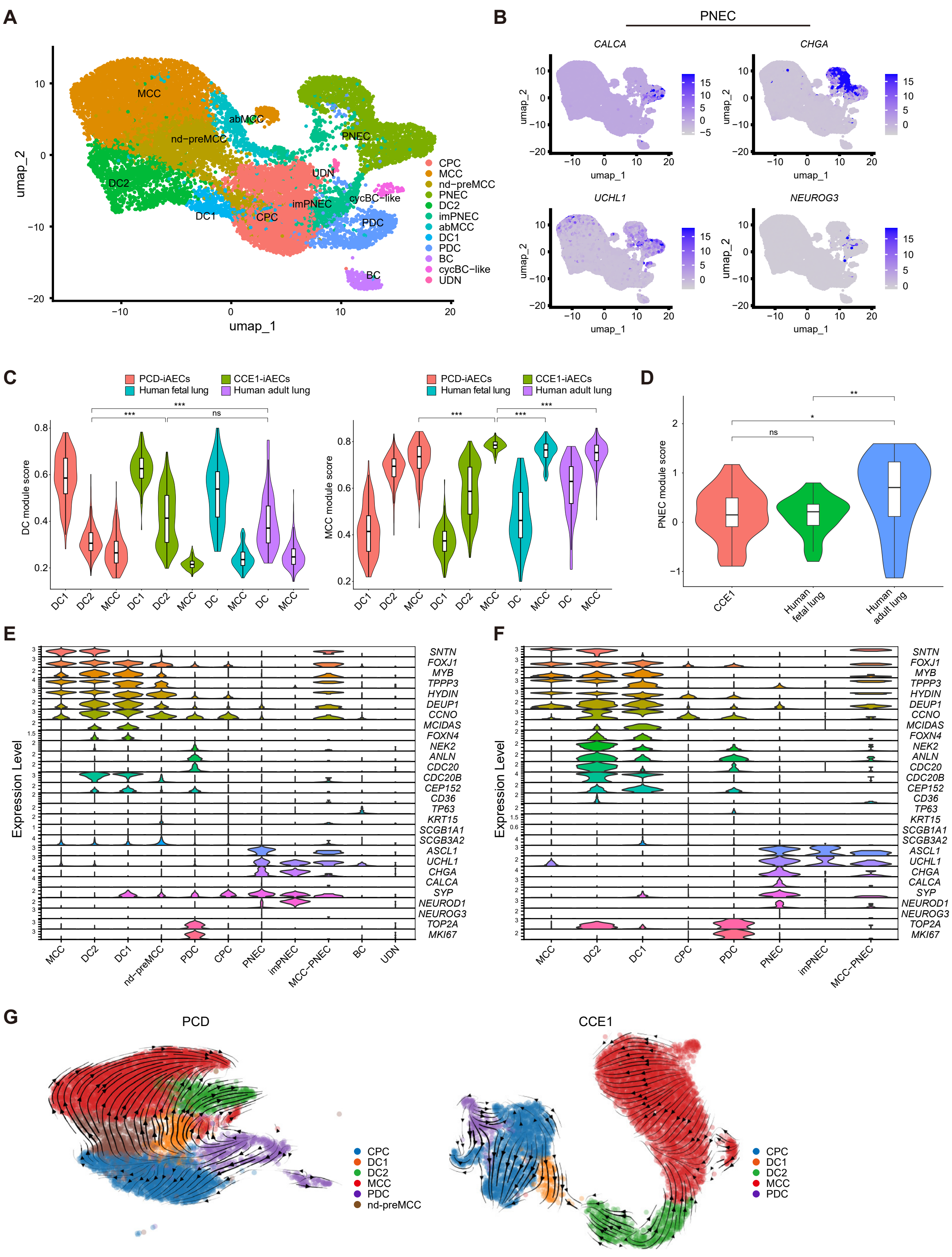

Figure S4. Additional scRNA-seq of PCD-iAECs and CCE1-iAECs, related to Figure 4.

(A) UMAP of integrated scRNA-seq derived from PCD- and CCE1-iAECs on ALI day 8.

(B) Feature plots of representative PNEC markers.

(C) Module score-based comparison of DC (left) and MCC (right) transcriptional programs among CCE1-iAECs, PCD-iAECs, human fetal AE cells (https://fetal-lung.cellgeni.sanger.ac.uk/scRNA.html; He et al., 2022), and human adult AE cells (GSE102580; Plasschaert et al., 2018). Statistical significance was assessed using Wilcoxon rank-sum test with Bonferroni correction. Selected biologically relevant between-group comparisons are indicated (*adjusted $P < 0.05$, **adjusted $P < 0.01$, ***adjusted $P < 0.001$; ns, not significant). Complete statistical results are provided in Tables S2 and S3.

(D) Module score–based comparison of PNEC transcriptional programs across CCE1-iAECs, human fetal AE cells (He et al., 2022), and human adult AE cells (GSE150674; Carraro et al., 2021). Statistical significance was determined using Wilcoxon rank-sum test: CCE1 *vs*. human fetal lung, not significant (adjusted $P = 0.779$); CCE1 *vs*. Human adult lung, *adjusted $P = 0.024$; human fetal lung *vs*. human adult lung, **adjusted $P = 0.020$.

(E, F) Violin plots of representative markers for MCCs, DCs, BCs, and PNECs, as well as *CD36* and proliferation markers in each cluster of PCD-iAECs (E) and CCE1-iAECs (F). Each dataset was clustered separately before integration.

(G) RNA velocity analysis of PCD-iAECs (left) and CCE1-iAECs (right) based on pre-integration scRNA-seq data. Velocity streamlines are overlaid on UMAP embeddings.

Supplemental Tables

Table S1. Statistical quantification of immunostained DCs and MCCs during ALI differentiation (mean ± SEM, %, n=3)

Cell population	Day 0	Day 4	Day 8	Day 12	Day 16
$CCNO^{+}NEK2^{+}$	0.00 ± 0.00	1.47 ± 0.23	4.93 ± 0.07	4.69 ± 0.13	4.04 ± 0.26
$CCNO^{+}NEK2^{-}$	0.00 ± 0.00	18.61 ± 0.82	4.02 ± 0.24	4.85 ± 0.21	2.80 ± 0.62
$CCNO^{-}NEK2^{+}$	0.00 ± 0.00	0.45 ± 0.14	1.96 ± 0.24	0.74 ± 0.15	0.87 ± 0.18
$CCNO^{-}NEK2^{-}$	100.00 ± 0.00	79.47 ± 0.75	89.10 ± 0.35	89.72 ± 0.43	92.29 ± 0.98
$FOXJ1^{+}NEK2^{+}$	0.00 ± 0.00	3.19 ± 0.28	6.82 ± 0.37	2.47 ± 0.38	5.08 ± 1.24
$FOXJ1^{+}NEK2^{-}$	0.17 ± 0.09	25.64 ± 1.73	17.56 ± 1.31	28.54 ± 0.50	57.57 ± 1.51
$FOXJ1^{-}NEK2^{+}$	0.00 ± 0.00	0.34 ± 0.03	0.46 ± 0.20	0.56 ± 0.16	0.61 ± 0.11
$FOXJ1^{-}NEK2^{-}$	99.83 ± 0.09	70.84 ± 2.02	75.16 ± 1.53	68.43 ± 1.00	36.07 ± 1.33

Table S2. Statistical comparison of DC and MCC clusters based on module score analysis

Module score name	Comparison type	Group1	Group2	padj	Significance
DC module score	within	PCD-iAECs_DC1	PCD-iAECs_DC2	2.53224E-75	***
DC module score	within	PCD-iAECs_DC1	PCD-iAECs_MCC	1.16656E-62	***
DC module score	within	PCD-iAECs_DC2	PCD-iAECs_MCC	2.81381E-18	***
DC module score	within	CCE1-iAECs_DC1	CCE1-iAECs_DC2	8.38606E-63	***
DC module score	within	CCE1-iAECs_DC1	CCE1-iAECs_MCC	1.87311E-65	***
DC module score	within	CCE1-iAECs_DC2	CCE1-iAECs_MCC	6.2977E-112	***
DC module score	within	HFL_DC	HFL_MCC	6.29512E-41	***
DC module score	within	HAL_DC	HAL_MCC	2.12942E-27	***
DC module score	between	PCD-iAECs_DC1	CCE1-iAECs_DC1	0.001540322	**
DC module score	between	PCD-iAECs_DC1	HFL_DC	0.000143031	***
DC module score	between	PCD-iAECs_DC1	HAL_DC	1.36187E-21	***
DC module score	between	CCE1-iAECs_DC1	HFL_DC	1.21154E-10	***
DC module score	between	CCE1-iAECs_DC1	HAL_DC	6.14582E-28	***
DC module score	between	HFL_DC	HAL_DC	2.47666E-09	***
DC module score	between	PCD-iAECs_DC2	CCE1-iAECs_DC2	2.15004E-44	***
DC module score	between	PCD-iAECs_DC2	HFL_DC	1.35107E-34	***
DC module score	between	PCD-iAECs_DC2	HAL_DC	1.36686E-09	***
DC module score	between	CCE1-iAECs_DC2	HFL_DC	4.74618E-11	***
DC module score	between	CCE1-iAECs_DC2	HAL_DC	0.100006002	ns
DC module score	between	HFL_DC	HAL_DC	1.98133E-09	***
DC module score	between	PCD-iAECs_MCC	CCE1-iAECs_MCC	1.08776E-24	***
DC module score	between	PCD-iAECs_MCC	HFL_MCC	5.04651E-07	***
DC module score	between	PCD-iAECs_MCC	HAL_MCC	0.001928841	**
DC module score	between	CCE1-iAECs_MCC	HFL_MCC	3.7958E-12	***
DC module score	between	CCE1-iAECs_MCC	HAL_MCC	1.46725E-19	***
DC module score	between	HFL_MCC	HAL_MCC	0.018454387	*

MCC module score	within	PCD-iAECs_DC1	PCD-iAECs_DC2	2.53224E-75	***
MCC module score	within	PCD-iAECs_DC1	PCD-iAECs_MCC	1.16656E-62	***
MCC module score	within	PCD-iAECs_DC2	PCD-iAECs_MCC	2.81381E-18	***
MCC module score	within	CCE1-iAECs_DC1	CCE1-iAECs_DC2	8.38606E-63	***
MCC module score	within	CCE1-iAECs_DC1	CCE1-iAECs_MCC	1.87311E-65	***
MCC module score	within	CCE1-iAECs_DC2	CCE1-iAECs_MCC	6.2977E-112	***
MCC module score	within	HFL_DC	HFL_MCC	6.29512E-41	***
MCC module score	within	HAL_DC	HAL_MCC	2.12942E-27	***
MCC module score	between	PCD-iAECs_DC1	CCE1-iAECs_DC1	0.001540322	**
MCC module score	between	PCD-iAECs_DC1	HFL_DC	0.000143031	***
MCC module score	between	PCD-iAECs_DC1	HAL_DC	1.36187E-21	***
MCC module score	between	CCE1-iAECs_DC1	HFL_DC	1.21154E-10	***
MCC module score	between	CCE1-iAECs_DC1	HAL_DC	6.14582E-28	***
MCC module score	between	HFL_DC	HAL_DC	2.47666E-09	***
MCC module score	between	PCD-iAECs_DC2	CCE1-iAECs_DC2	2.15004E-44	***
MCC module score	between	PCD-iAECs_DC2	HFL_DC	1.35107E-34	***
MCC module score	between	PCD-iAECs_DC2	HAL_DC	1.36686E-09	***
MCC module score	between	CCE1-iAECs_DC2	HFL_DC	4.74618E-11	***
MCC module score	between	CCE1-iAECs_DC2	HAL_DC	0.100006002	ns
MCC module score	between	HFL_DC	HAL_DC	1.98133E-09	***
MCC module score	between	PCD-iAECs_MCC	CCE1-iAECs_MCC	1.08776E-24	***
MCC module score	between	PCD-iAECs_MCC	HFL_MCC	5.04651E-07	***
MCC module score	between	PCD-iAECs_MCC	HAL_MCC	0.001928841	**
MCC module score	between	CCE1-iAECs_MCC	HFL_MCC	3.7958E-12	***
MCC module score	between	CCE1-iAECs_MCC	HAL_MCC	1.46725E-19	***
MCC module score	between	HFL_MCC	HAL_MCC	0.018454387	*

SUPPLEMENTAL EXPERIMENTAL PROCEDURES

Human adult and fetal lung tissues

We obtained human adult lung tissues from residual non-tumorous tissues resected during surgery for lung cancer. Three donors (two females aged 58 and 78 years and one 78-year-old male) provided written informed consent for the use of their tissues. Resected adult lung tissues were fixed in 4% paraformaldehyde (PFA), cryoprotected, embedded in optimal cutting temperature (OCT) compound, and stored at −80°C. Frozen human fetal lung tissues were purchased from DV Biologics (18.5 weeks of gestation, PP001-FS, Lot. 102508RH).

Generation and maintenance of patient-derived iPSCs

Peripheral blood mononuclear cells (PBMCs) were obtained from patients with PCD harboring compound heterozygous *CCNO* variants. We reprogrammed PBMCs into iPSCs as described by Okita et al. (2011). We seeded 3×10^6 PBMCs /well in 6-well plates (Corning, NY, USA) with 1.5 mL of StemSpan-Animal Component-Free (ACF) medium (09860; STEMCELL Technologies, Vancouver, BC, Canada). The cells were then incubated for 5 days in medium supplemented with 100 ng/mL each of Stem Cell Factor (SCF; 255-SC), thrombopoietin (TPO; 288-TP), FLT3L (308-FK), and IL-6 (206-IL) as well as 20 ng/mL IL-3 (203-IL; all from R&D Systems, Minneapolis, MN, USA). Putative iPSC colonies were induced by transfecting the cells with human iPS generation episomal vector mix (3673; Takara Bio, Kusatsu, Japan) containing cDNAs for OCT3/4, SOX2, KLF4, L-MYC, LIN28, mp53-DD, and EBNA1. Cells were incubated until putative iPSC colonies emerged. These were manually selected as described by the manufacturer. Isolated iPSC colonies were maintained in StemFit AK02N (AJ100; Ajinomoto, Tokyo, Japan) on plates coated with iMatrix-511 silk (892021; Nippi, Tokyo, Japan) and then cryopreserved in STEM-CELLBANKER (CB045; Takara Bio). The colonies were thawed, recovered in StemFit AK02N medium, maintained, and passaged in mTeSR Plus medium (ST-100-0276; STEMCELL Technologies) before differentiation. Cells were maintained in culture medium supplemented with penicillin–streptomycin. Mycoplasma contamination was routinely monitored using the MycoAlert Mycoplasma Detection Kit (LT07-318; Lonza Group AG, Basel, Switzerland) as described by the manufacturer. We analyzed the differentiation of the iPSC clone CiRA01103-11, which is hereafter referred to as PCD-iPSCs. Trilineage differentiation of PCD-iPSCs into endoderm, mesoderm, and ectoderm was performed using STEMdiff Trilineage Differentiation Kit (ST-05230; STEMCELL Technologies) as described by the manufacturer. Expression of pluripotency markers in the three germ layers was assessed using flow cytometry.

Gene correction of PCD-iPSCs

Genes were corrected using the CRISPR/Cas9 system and Lipofectamine Stem Transfection Reagent (STEM00015; Thermo Fisher Scientific, Waltham, MA, USA) as described by the manufacturer. We seeded PCD-iPSCs at a density of 5×10^5 cells/well on iMatrix-coated 6-well plates and incubated them in mTeSR Plus with 10 μM Y-27632 (HY-10583; MedChemExpress, Monmouth Junction, NJ, USA). One day later, the cells were transfected with 2 μg/well of the following plasmid vectors (Li et al., 2016): a Cas9 expression vector, a single-guide RNA (sgRNA) expression vector targeting the variant locus, and a donor vector (constructed using VectorBuilder) containing the wild-type sequence and neomycin resistance cassette. The plasmids were mixed with Lipofectamine Stem in Opti-MEM (31985062; Gibco, Thermo Fisher Scientific) to form transfection complexes that were added directly to the wells. On the following

day, the medium was replaced with fresh mTeSR Plus. G418 (10131027; Gibco) selection was initiated several days after transfection, depending on cell confluence and recovery, and maintained for an appropriate duration to isolate resistant colonies. After limiting dilutions, individual colonies were manually selected using a microscope, expanded in separate wells, and screened for gene correction using genomic DNA extraction followed by PCR amplification of the target locus. To excise the neomycin resistance cassette, 1 × 10^6 cells were electroporated with 5 µg of a Cre expression plasmid using a NEPA21 electroporator (Nepa Gene Co., Ltd., Ichikawa City, Japan). Cells were then allowed to recover without selection, and subjected to limiting dilution to isolate single-cell clones. Excision was confirmed using PCR and Sanger sequencing, and the following independently corrected clones were obtained: CCE1 (clone 71-16), in which the variant in exon 1 was corrected, and CCE3 (clone 42-2), in which the variant in exon 3 was corrected (hereafter referred to as CCE1- and CCE3-iPSCs, respectively). Predicted off-target sites with up to three base-pair mismatches were identified using the GGGenome database (https://gggenome.dbcls.jp/en/) and analyzed by Sanger sequencing. Table S3 shows the predicted off-target sites, and Table S4 shows the primer sequences used for amplification and sequencing.

Karyotype analysis

The PCD-, CCE1-, and CCE3-iPSC lines were karyotyped using G-banding at Nihon Gene Research Laboratories, Inc., Sendai, Japan.

Short tandem repeat (STR) profiling

We verified the genetic identities of patient-derived PBMCs, PCD-iPSCs, and gene-corrected iPSC lines (CCE1 and CCE3) by STR profiling using the PowerPlex 16 HS System (Promega, Madison, WI, USA).

Generation of a rat monoclonal antibody against CPM

Wistar rats (n = 4) were intraperitoneally immunized with 50 µg of recombinant human carboxypeptidase M (CPM; CPM-H5222; Acro Biosystems, Newark, DE, USA) emulsified with four doses of adjuvant at 2-week intervals, followed by a final 200 µg booster injection without adjuvant. Antibody responses were initially screened by ELISA using recombinant CPM protein, followed by flow cytometry using H2228 cells (CRL-5935; ATCC, Manassas, VA, USA) that endogenously express CPM. Based on these results, selected rats were used to generate hybridomas. Splenocytes from selected rats were fused with a mouse myeloma cell line using polyethylene glycol (PEG), and hybridomas were selected in a selective medium. Approximately 10 days after fusion, the culture supernatants were screened using ELISA and flow cytometry to identify clones that produced CPM-specific antibodies. Positive clones underwent two rounds of limiting dilutions. Clones were initially screened at each stage using an ELISA to identify CPM-reactive antibodies, and then antigen specificity was evaluated using flow cytometry in H2228 cells and their CPM-knockout counterparts (CPM-KO H2228). We generated CPM-KO H2228 cells by electroporating two plasmids encoding Cas9 and an sgRNA targeting CPM using the same vector system as that used for *CCNO* gene correction in iPSCs and the NEPA21 electroporator. The selected hybridomas were expanded in a medium containing serum and then transferred to serum-free medium for finally producing antibodies. Supernatants were subjected to IgG purification by Protein G affinity chromatography. Monoclonal antibody production and purification were performed at Immuno-Biological Laboratories Co., Ltd. (IBL; Fujioka, Japan), and flow cytometry-based evaluations were conducted in house.

Differentiation of human iPSCs into lung progenitor cells

Human iPSCs were differentiated into lung progenitor cells as described by Gotoh et al. (2014), Konishi et al. (2016), and Yamamoto et al. (2017). The undifferentiated iPSC lines, B2-3 (p70–80), 585A1 (p40–50) (Okita et al., 2013), CiRA01103-11 (p8–20), and CCE1 71-16 (p25–30), were differentiated into definitive endoderm on plates coated with Geltrex (A14113322; Thermo Fisher Scientific) in RPMI1640 medium (30264-56; Nacalai Tesque, Kyoto, Japan) supplemented with 100 ng/mL activin A (GF-001-050L; ALI Co., Ltd., Gifu, Japan), 1 mM CHIR99021 (AXN-AXON1386-25; AXON Medchem, Gronigen, Netherlands), and 2% B27 supplement (17504-001; Thermo Fisher Scientific). On day 0, 10 μM Y-27632 was added, and sodium butyrate (193-01522; Wako, Osaka, Japan) was added on days 1, 2, and 4. The cells were incubated on days 6–9 in anteriorization medium containing 10 μM SB431542 (198-16543; Wako) and 100 ng/mL Noggin (6057-NG-01M; R&D Systems). The cells were incubated on days 10–13 in ventralization medium containing 3 μM CHIR99021, 20 ng/mL BMP4 (314-BP-01M; R&D Systems), and all-trans retinoic acid (ATRA; R2625; Sigma-Aldrich, St. Louis, MO, USA) at optimized concentrations (0.5 μM for PCD- and CCE1-iPSCs). For airway epithelial (AE) differentiation via direct air–liquid interface (ALI) protocol, cells were cultured in CFKD preconditioning medium containing 3 μM CHIR99021, 10 ng/mL each of FGF10 (100-26) and KGF (100-19-250UG; both from PeproTech, Cranbury, NJ, USA), and 20 μM DAPT (049-33583; Wako) on days 14–20. The medium was changed every 2 days. We generated iAECs by purifying NKX2-1^{+} lung progenitor cells using CPM antibody-based, magnetically activated cell sorting (MACS) on days 14 and 21 for the 3D- and direct ALI protocols, respectively, as described below.

Differentiation of lung progenitor cells into AE cells via 3D Culture

We applied the 3D-ALI differentiation protocol as described by Konishi et al. (2016). We isolated CPM^{+} cells on day 14 of the lung progenitor cell differentiation protocol using a mouse anti-human CPM monoclonal antibody (014-27501; Wako) and anti-mouse IgG microbeads (130-048-401; Miltenyi Biotec, Bergisch Gladbach, Germany) with LS columns (130-042-401; Miltenyi Biotec). The sorted cells were resuspended in Step 4 medium containing 3 μM CHIR99021, 100 ng/mL FGF10, and 10 μM Y-27632, and mixed with growth factor-reduced Matrigel (354230; Corning) at a 1:1 ratio. Suspensions containing 5.0 × 10^{5} cells/cm^{2} were poured onto cell culture inserts (353095, 353180; Corning) and cultured under 3D conditions for 14 days in Step 4 medium. The medium in the lower chamber was replaced with PneumaCult-ALI Maintenance Medium supplemented with 10 μM Y-27632 and 10 μM DAPT. PneumaCult-ALI Maintenance Medium was prepared by supplementing PneumaCult-ALI medium (ST-05001; STEMCELL Technologies) with 4 μg/mL heparin (17513-41; Nacalai Tesque) and 1 μM hydrocortisone (H0888; Sigma-Aldrich). The cells were cultured for an additional 14 days, and the resulting spheroid organoids were then dissociated and seeded onto Geltrex-coated cell culture inserts (9.4 × 10^{5} cells/cm^{2}) for ALI culture. Cells were maintained in PneumaCult-ALI Maintenance Medium supplemented with 10 μM Y-27632 and 10 μM DAPT for 20 days.

Differentiation of lung progenitor cells into AE cells via direct ALI seeding

The direct ALI differentiation protocol was performed as described by Yin et al. (2021). We isolated CPM^{+} cells on day 21 of the lung progenitor cell protocol using a rat anti-human CPM antibody (generated in-house) and anti-rat kappa microbeads (130-047-401) with an autoMACS Pro Separator (both from Miltenyi Biotec). The sorted cells (1.8 × 10^{6} cells/cm^{2}) were seeded

onto iMatrix-coated cell culture inserts for ALI culture using the PneumaCult-ALI Maintenance Medium supplemented with 10 μM Y-27632. After approximately 2 days of culture, once the cells reached confluence, the medium was replaced with the PneumaCult-ALI Maintenance Medium supplemented with 10 μM Y-27632 and 10 μM DAPT, and this time point was designated as ALI day 0. The medium was replaced weekly.

Flow cytometry and fluorescence-activated cell sorting (FACS)

Suspensions of live single cells were washed with 1% BSA/PBS and stained with primary antibodies at 4°C for 15 min. The cells were washed twice with 1% BSA/PBS and then incubated with secondary antibodies at 4°C for 15 min. The cells were again washed twice and incubated with propidium iodide (54722; Nacalai Tesque) for live/dead discrimination. For all iAEC analyses, $EPCAM^+$ epithelial cells were gated within the live cell population, and marker expression was assessed within the $EPCAM^+$ population. For intracellular staining of undifferentiated iPSCs and trilineage-differentiated cells, we fixed and permeabilized single-cell suspensions in Fixation and Permeabilization Solution (554722; BD Biosciences, Franklin Lakes, NJ, USA) at 25°C for 20 min. After two washes with Perm/Wash Buffer (554723; BD Biosciences), we incubated them with primary antibodies at 4°C for 30 min. The cells were again washed twice and then incubated with secondary antibodies at 4°C for 30 min. After two final washes with 1% BSA/PBS, the cells were resuspended in 1% BSA/PBS without propidium iodide. Flow cytometry and cell sorting were performed using a FACS Melody Cell Sorter (BD Biosciences). Table S5 shows the antibodies used in this study.

CD36-based re-plating analysis

We isolated $CD36^+$ and $CD36^-$ cells by FACS on ALI day 8 of the direct ALI differentiation protocol, and seeded them onto iMatrix-coated cell culture inserts at a density of 1.4×10^6 cells/cm² for ALI culture. The cells were cultured in PneumaCult-ALI Maintenance Medium supplemented with 10 μM Y-27632. After 3 days of culture, when the cells had attached and reached confluence, the medium was replaced with PneumaCult-ALI Maintenance Medium supplemented with 10 μM Y-27632 and 10 μM DAPT. This time point was designated as ALI day 0. On ALI day 2, cells on the membrane were fixed and analyzed by immunofluorescence staining.

Immunofluorescence staining

Cells incubated on cell culture insert membranes were fixed with 4% PFA for 30 min, followed by permeabilization with 0.2% Triton X-100 in PBS for 30 min. Depending on the primary antibody, the cells were fixed and permeabilized with ice-cold methanol overnight. After blocking nonspecific binding of secondary antibodies for 1 h with 5% donkey serum (S30-100ML; Millipore, Burlington, MA, USA) in PBS at room temperature, samples were incubated with primary antibodies diluted in blocking buffer overnight at 4°C. After three washes with PBS, the samples were incubated with appropriate secondary antibodies for 30 min at room temperature in the dark. Human adult lung tissue sections were permeabilized with 0.2% Triton X-100 in PBS for 30 min before immunostaining. Samples of fetal human lung tissue sections were fixed with 4% PFA for 30 min and subsequently permeabilized with 0.2% Triton X-100 in PBS for 30 min before immunostaining. Thereafter, blocking and primary and secondary antibody incubations were performed under the same conditions as those applied to the cells cultured on the insert membranes. For immunostaining of cytospin slides, cells were cytocentrifuged onto glass slides, fixed with 4% PFA for 15 min, permeabilized with 0.2% Triton X-100 in PBS for 15

min, then incubated with primary and secondary antibodies for 20 min each. Nuclei were counterstained with Hoechst 33342 (H342; Dojindo Laboratories, Kumamoto, Japan). Antibody information, including the fixation conditions and dilution rate for each antibody used in this study, is listed in Table S5. Images were acquired using an FV3000 confocal laser scanning microscope (Evident Inc., Tokyo, Japan) and a BZ-X710 microscope (Keyence, Tokyo, Japan). Z-stack images acquired using the FV3000 were processed to generate maximum intensity projections and orthogonal views, which were quantified using ImageJ software (NIH, Bethesda, MD, USA).

Quantitative Real-time PCR (RT-qPCR)

Total RNA was extracted from the cultured cells using an RNeasy Micro Kit (74004; Qiagen, Hilden, Germany). cDNA was prepared from 5 to 80 ng of total RNA per sample with SuperScript III Reverse Transcriptase (18080044; Thermo Fisher Scientific), amplified using Power SYBR Green PCR Master Mix (4368708; Applied Biosystems, Waltham, MA, USA), and quantified using a QuantStudio 3 (Applied Biosystems). Gene expression was normalized to β-actin expression levels. Exogenous control RNA from human adult trachea (29 years old; R1234160-50; BioChain, Newark, CA, USA; Lot. B803066) was used to calculate relative gene expression. Primers used are listed in Table S6.

RNA preparation for bulk RNA-seq

$CD36^+$ and $CD36^-$ cells were isolated using flow cytometry on ALI day 12 following direct ALI differentiation of CPM^+ cells derived from B2-3 SFTPC-GFP reporter hiPSCs. The B2-3 line was generated from the 201B7 hiPSC line, originally established from a healthy donor (Takahashi et al., 2007; Gotoh et al., 2014). Total RNA from $CD36^+$ and $CD36^-$ cells was isolated using the RNeasy Micro Kit (74004; Qiagen) following the manufacturer's instructions. The RNA integrity of each sample was assessed using an Agilent 2100 BioAnalyzer, with all samples showing RNA integrity at 85s (RIN) exceeding 9.5. To compare $CD36^+$ and $CD36^-$ cell populations, 10 ng of total RNA was used for RNA-seq library preparation using Illumina Stranded Total RNA Prep Ligation with Ribo-Zero Plus (Cat. 20040525) according to the manufacturer's instructions. Sequencing was performed on Illumina NextSeq 2000 platform in the paired-end mode. The FASTQ files were generated using bcl2fastq-2.20.

Bulk RNA-seq and differential gene expression analysis

Adapter sequences and low-quality bases were trimmed from raw reads using Cutadapt v4.6. The trimmed reads were mapped to the human reference genome sequence (hg38) and reporter (EGFP) sequence using STAR v2.7.11a (Dobin et al., 2013) with the GENCODE (release 32, GRCh38.p13) (Frankish et al., 2019) GTF file. Raw counts were calculated using HTSeq-count v2.0.5 using a GENCODE GTF file. Gene expression is shown as transcripts per million (TPM). Genes in all samples with an average TPM < 0.5 were excluded from downstream analyses. The resulting expression matrix was log-transformed to log2(TPM+1) and normalized using row-wise Z-score scaling. Gene expression profiles were visualized by hierarchical clustering and heatmaps were generated using the heatmap.2 function in R. Hierarchical clustering was conducted using the Ward method with Euclidean distance (Murtagh and Legendre, 2014), and then PCA was performed using the 300 most variable genes across all samples. We generated PCA plots using ggplot2, and each point represents a biological replicate colored by sample ID and shaped by CD36 status. Principal components (PC1 and PC2) explained 95.3% and 1.75% of variance, respectively. Differential gene expression

between CD36$^+$ and CD36$^-$ cell populations was analyzed using Wald tests implemented in DESeq2, with Benjamini–Hochberg correction for multiple tests (Benjamini and Hochberg, 1995; Love et al., 2014). Genes with $|\log_2$ fold change$| > 1$ and adjusted $P < 0.05$ were considered significantly and differentially expressed. Volcano plots were generated using the EnhancedVolcano package in R and selected genes of interest, including multiciliogenesis and airway lineage markers, were individually visualized to highlight their expression profiles. Gene Ontology (GO) enrichment analysis of differentially upregulated genes in CD36$^+$ cells was performed using the clusterProfiler R package (Yu et al., 2012). Gene IDs were converted to Entrez IDs using the org.Hs.eg.db database, and enrichment was assessed using biological processes in the GO categories. The enriched terms were visualized using bar and dot plots and tab-delimited result tables were exported.

scRNA-seq of iAECs

scRNA-seq was performed using AE cells derived from human iPSCs, including the 201B7, PCD-iPSC, and CCE1-iPSC lines, that were all differentiated under ALI conditions. The 201B7 cell line was processed using the 3D-ALI differentiation protocol as described by Konishi et al. (2016), which included the addition of a Notch inhibitor to promote MCC differentiation and focus on multiciliogenesis. Airway organoids were initially generated in 3D cultures and then replated into an ALI system on day 42. Single-cell suspensions were collected on day 62. In contrast, CPM$^+$ cells from PCD- and CCE1-iPSC lines were isolated and directly seeded into ALI cultures as described above (direct ALI differentiation protocol), with single-cell suspensions collected on day 8 after the initiation of ALI differentiation. Single-cell suspensions were prepared *via* enzymatic dissociation. Cells cultured on inserts were rinsed with warm PBS, incubated with 0.5 mM EDTA in PBS at 37°C for 10 min, followed by Accutase at 37 °C for 20 min. Dissociation was facilitated by gentle pipetting followed by passing the suspension through a 40 μm cell strainer to remove cell clumps. Single-cell libraries were constructed using the Chromium platform (10x Genomics) and Single Cell 3′ Reagent Kits v3.1 (for 201B7) or v4.0 (for PCD- and CCE1-iPSC) as described by the manufacturer. Sequencing data were processed using Cell Ranger (v8.0.1) for demultiplexing, alignment to the GRCh38 reference genome (refdata-gex-GRCh38-2020-A, including reporter sequences), and generation of gene count matrices. To mitigate the influence of ambient RNA contamination, count matrices were corrected using SoupX (v1.6.2) (Young and Behjati, 2020). The cleaned matrices were imported into Seurat (v5.0.0), and then Seurat objects were constructed by filtering cells with ≥200 genes and genes expressed in ≥3 cells. Cells with >15% mitochondrial RNA and <200 or >10,000 detected genes were excluded. Mitochondrial reads were quantified using the PercentageFeatureSet with the pattern "^MT-." Gene expression data were normalized using LogNormalize (scaling factor = 10,000) and highly variable features (top 2,000) were identified using variance-stabilizing transformation. All genes were scaled before dimensionality reduction using PCA. We used FindNeighbors and FindClusters to identify groups of similar cells. UMAP embeddings were generated for visualization using RunUMAP. To assess potential doublet contamination, DoubletFinder was applied to each dataset using parameters optimized for the estimated doublet rate, and the predicted doublets were removed (McGinnis et al., 2019). Initial clusters were manually annotated based on the expression of canonical marker genes in each cell lineage. A subset of epithelial clusters was extracted for refined analysis of 201B7-derived iAECs, and then re-clustered using PCA, FindNeighbors, and FindClusters. The UMAPs were regenerated using the adjusted parameters. Differential gene expression was evaluated using FindAllMarkers with a $\log_2$ fold change cutoff > 0.25 and expression in >25% of cells. Additional

expression was visualized using VlnPlot, DotPlot, and FeaturePlot software. We enabled a direct comparison between PCD- and CCE1-iAECs by analyzing integration using Seurat (v5.0.0) with SCTransform-based normalization. Seurat objects for each dataset (PCD and CCE1) were normalized independently using SCTransform with the top 2,000 variable features. Subsequently, integration features were selected using SelectIntegrationFeatures, and integration anchors were identified using FindIntegrationAnchors with SCT normalization. Datasets were integrated using IntegrateData to create a single Seurat object containing both conditions. Dimensionality was reduced using PCA, followed by neighborhood graph construction using FindNeighbors, clustering with FindClusters, and visualization with RunUMAP. Condition-specific comparisons were visualized by splitting UMAPs and violin plots into experimental groups (split.by = "condition"), which allowed direct evaluation of cluster composition between PCD and CCE1 samples. Pseudotime trajectories were analyzed using Monocle 3 (Cao et al., 2019), and pseudotime dynamics were visualized using plot_cells with Viridis color gradients. All data were analyzed using the following R (v4.3.1) packages: Seurat (v5.0.0), monocle3, SoupX, DoubletFinder, sctransform, patchwork, ggplot2, tidyverse, and ROCR.

Module score–based comparisons of MCCs, DCs, and PNECs among iPSC-derived, fetal, and adult human AE cells

We evaluated the fidelity of iPSC-derived AE cells (iPSC-AECs) to native human AE using single-cell transcriptomic comparisons among multiple developmental and experimental contexts. All single-cell comparisons were performed to evaluate similar transcriptional states among datasets rather than to infer donor-level population differences.

We used GSE102580 (Plasschaert et al., *Nature*, 2018) for DCs and MCCs and GSE150674 (Carraro et al., *Nature Medicine*, 2021) for PNECs to analyze single-cell RNA-seq datasets from CCE1-iAECs derived from a gene-corrected hiPSC line harboring a *CCNO* variant; PCD-iAECs derived from a *CCNO*-variant patient hiPSC line; epithelial cell populations from human fetal lung (https://fetal-lung.cellgeni.sanger.ac.uk/scRNA.html; He et al., *Cell*, 2022); and epithelial cell populations from adult human lungs.

We used the cell-type annotations provided by the original authors for the fetal lung dataset and extracted ciliated cell, DC, and PNEC populations without re-deriving cluster identities for adult lung comparisons. The Carraro et al. dataset was specifically used for PNEC analyses because the data detected rare PNECs in adult human airways, enabling meaningful comparisons with iPSC-derived and fetal PNEC populations. Each dataset was independently processed using Seurat v5.0.0 in R (v4.3.2). Cells in each dataset were filtered based on mitochondrial RNA content as well as gene and UMI counts using dataset-specific thresholds. Normalization, variable feature selection, PCA, and UMAP embedding were performed according to the standard Seurat workflow and to enable cross-sample comparisons of epithelial differentiation states. We quantified transcriptional programs associated with MCCs, DCs, and PNECs using curated gene signatures. The deuterosomal signatures were *DEUP1, CDC20B, FOXN4, GMNC, MCIDAS, MYB, PLK4, CCNO*, and additional centriole biogenesis-related genes. The MCC signatures were *FOXJ1, TPPP3, SNTN, RSPH1*, and *DNAH5*. For the PNEC analysis, a neuroendocrine signature comprising *ASCL1, SYP, CHGA*, and *CALCA* was used.

The module scores were computed using Seurat's AddModuleScore function (Tirosh et al., 2016) and stored in metadata as S_Deutero, S_MCC, and S_PNEC. Because deuterosomal

and multiciliated differentiation programs represent opposing states along a shared differentiation axis, their relative state assignments were further quantified by deriving probabilistic class scores, P_Deutero and P_MCC, using a two-class softmax transformation to facilitate the relative interpretation of opposing transcriptional programs. In contrast, PNECs represent a lineage-distinct epithelial population rather than a transitional state along the multiciliated differentiation axis. Therefore, PNEC identity was evaluated using absolute module scores (S_PNEC) without probabilistic normalization.

We specifically compared PNECs in CCE1-iAECs and human fetal and adult lungs. Although CCE1-iAECs retain a *CCNO* exon 3 variant, they represent a functionally corrected line. Because PNECs are unlikely to depend on CCNO-mediated centriole amplification, we excluded PCD-iAECs from PNEC comparisons to avoid confounding disease-specific effects that were irrelevant to PNEC biology.

For comparative analyses, we downsampled cells to equal numbers across conditions before visualization and statistical tests to minimize bias arising from unequal cell numbers. Violin and box plots were generated using ggplot2, with faceting by dataset ("CCE1-iAECs," "PCD-iAECs," "Human Fetal Airway," and "Human Adult Airway"). We statistically compared module scores using two-sided Wilcoxon rank-sum tests. We adjusted *P* values for multiple pairwise comparisons using the Benjamini–Hochberg false discovery rate (FDR) correction. Adjusted *P* values are shown unless otherwise stated.

RNA Velocity Analysis

RNA velocity analysis was performed using scVelo (v0.3.2) in Python. Processed scRNA-seq datasets (PCD and CCE1) were first analyzed in R (Seurat v5.0.0) for quality control, dimensionality reduction, clustering, and cell-type annotation. To integrate these R-based annotations with spliced/unspliced matrices derived from BAM files, we imported the Seurat-derived UMAP coordinates and cluster annotations and aligned them with the corresponding loom files from Velocyto.

Electron microscopy

Matrigel-embedded organoids were fixed at room temperature for 30 min in 0.1 M phosphate buffer (pH 7.4) containing 2.5% glutaraldehyde, 4% PFA, and 1% tannic acid. After three rinses in 0.1 M phosphate buffer (pH 7.4) for 20 min, the samples were fixed in 1% osmium tetroxide (25727-01; Nacalai Tesque) for 2 h, dehydrated through a graded ethanol series, and embedded in pure Epon, as described by Konishi et al. (2016). Ultrathin sections were sliced using an ultramicrotome (UC6; Leica Microsystems, Wetzlar, Germany), stained with uranyl acetate and lead citrate, and analyzed using a Hitachi H-7650 transmission electron microscope (Hitachi, Tokyo, Japan).

SUPPLEMENTAL REFERENCES

Benjamini, Y., & Hochberg, Y. (1995). Controlling false discovery rate: A practical and powerful approach to multiple testing. *J. R. Stat. Soc. Series B Stat. Methodol.* 57, 289–300.

Cao, J., Spielmann, M., Qiu, X., Huang, X., et al. (2019). The single-cell transcriptional landscape of mammalian organogenesis. *Nature* 566, 496–502.

Chen, S., Zhou, Y., Chen, Y., Gu, J. (2018). fastp: ultrafast all-in-one FASTQ preprocessor. *Bioinformatics* 34, i884–i890.

Dobin, A., Davis, C.A., Schlesinger, F., Drenkow, J., Zaleski, C., Jha, S., Batut, P., Chaisson, M., and Gingeras, T.R. (2013). STAR: Ultrafast Universal RNA-seq Alignment *Bioinformatics* 29, 15–21.

Frankish A, Diekhans M. Ferreira, A.-M. Johnson, R. Jungreis, I. Loveland, J. Mudge, J.M. Sisu, C. Wright, J. Armstrong, J. et al. (2019). GENCODE reference annotation of human and mouse genomes. *Nucleic Acids Res.* 47, D766–D773.

Li and Dewey (2011). RSEM: accurate transcript quantification from RNA-Seq data with or without a reference genome. *BMC Bioinformatics* 12, 323.

Li, H.L., Gee, P., Ishida, K., Hotta, A. (2016). Efficient genomic correction methods for human iPS cells using the CRISPR-Cas9 system. *Methods* 101, 27–35.

Love, M.I., Huber, W., Anders, S. (2014). Moderate estimation of fold-change and dispersion of RNA-seq data using DESeq2. *Genome Biol.* 15, 550.

McGinnis, C. S., Murrow, L. M., Gartner, Z. (2019). DoubletFinder: Doublet detection in single-cell RNA sequencing data using artificial nearest neighbors. *Cell Syst.* 8, 329–337.e4.

Murtagh, F., & Legendre, P. (2014). Ward's hierarchical agglomerative clustering method: Which algorithms implement Ward's criteria? *J. Classif.* 31, 274–295.

Okita, K., Matsumura, Y., Sato, Y., Okada, A., Morizane, A., Okamoto, S., Hong, H., Nakagawa, M., Tanabe, K., Tezuka, K. et al. (2011). A more efficient method for generating integration-free human iPS cells. *Nat. Methods* 8, 409–412.

Okita, K., Yamakawa, T., Matsumura, Y., Sato, Y., Amano, N., Watanabe, A., Goshima, N., Yamanaka, S. (2013). An efficient nonviral method to generate integration-free human-induced pluripotent stem cells from cord blood and peripheral blood cells. Stem Cells 31, 458-466.

Takahashi, K., Tanabe, K., Ohnuki, M., Narita, M., Ichisaka, T., Tomoda, K., and Yamanaka, S. (2007). Induction of pluripotent stem cells from adult human fibroblasts using the defined factors. *Cell* 131, 861–872.

Yamamoto, Y., Gotoh, S., Korogi, Y., et al. (2017). Long-term expansion of human iPSC-derived alveolar stem cells into organoids. *Nat. Methods* 14, 1097–1106.

Yin, X., Riva, L., Pu, Y., et al. (2021). MDA5 Governs the Innate Immune Response to SARS-CoV-2 in Lung Epithelial Cells. *Cell Rep.* 34, 108628.

Young, M.D., and S. (2020). SoupX removes ambient RNA contamination from droplet-based single-cell RNA sequencing data. *GigaScience* 9, giaa151.

Yu, G., Wang, L.G., Han, Y., and He, Q.Y. (2012). ClusterProfiler: R package for comparing biological themes among gene clusters. *OMICS* 16, 284–287.

Carraro, G., Langerman, J., Sabri, S., Lorenzana, Z., Purkayastha, A., Zhang, G., Konda, B., Aros, C.J., Calvert, B.A., Szymaniak, A., et al. (2021). Transcriptional analysis of cystic fibrosis airways at single-cell resolution reveals altered epithelial cell states and composition. *Nature Medicine* 27,

806-814.

Tirosh, I., Izar, B., Prakadan, S.M., Wadsworth, M.H., 2nd, Treacy, D., Trombetta, J.J., Rotem, A., Rodman, C., Lian, C., Murphy, G., et al. (2016). Dissecting the multicellular ecosystem of metastatic melanoma by single-cell RNA-seq. *Science* 352, 189-196.
